# Long non-coding RNA DLEU2 drives EMT and glycolysis in endometrial cancer through HK2 by competitively binding with miR-455 and by modulating the EZH2/miR-181a pathway

**DOI:** 10.1186/s13046-021-02018-1

**Published:** 2021-06-26

**Authors:** Peixin Dong, Ying Xiong, Yosuke Konno, Kei Ihira, Noriko Kobayashi, Junming Yue, Hidemichi Watari

**Affiliations:** 1 Department of Obstetrics and Gynecology, Hokkaido University School of Medicine, Hokkaido University, Sapporo, 0608638 Japan; 2 Department of Gynecology, State Key Laboratory of Oncology in South China, Sun Yat-sen University Cancer Center, Guangzhou, 510060 China; 3 Department of Pathology and Laboratory Medicine, University of Tennessee Health Science Center, Memphis, TN 38163 USA; 4 Center for Cancer Research, University of Tennessee Health Science Center, Memphis, TN 38163 USA

**Keywords:** HK2, FAK, ERK1/2 signaling, DLEU2, Long noncoding RNA, LncRNA, MicroRNA, EMT, Aerobic glycolysis, Endometrial cancer

## Abstract

**Background:**

Epithelial-to-mesenchymal transition (EMT) and aerobic glycolysis are fundamental processes implicated in cancer metastasis. Although increasing evidence demonstrates an association between EMT induction and enhanced aerobic glycolysis in human cancer, the mechanisms linking these two conditions in endometrial cancer (EC) cells remain poorly defined.

**Methods:**

We characterized the role and molecular mechanism of the glycolytic enzyme hexokinase 2 (HK2) in mediating EMT and glycolysis and investigated how long noncoding RNA DLEU2 contributes to the stimulation of EMT and glycolysis via upregulation of HK2 expression.

**Results:**

HK2 was highly expressed in EC tissues, and its expression was associated with poor overall survival. Overexpression of HK2 effectively promoted EMT phenotypes and enhanced aerobic glycolysis in EC cells via activating FAK and its downstream ERK1/2 signaling. Moreover, microRNA-455 (miR-455) served as a tumor suppressor by directly interacting with *HK2* mRNA and inhibiting its expression. Furthermore, DLEU2 displayed a significantly higher expression in EC tissues, and increased DLEU2 expression was correlated with worse overall survival. DLEU2 acted as an upstream activator for HK2-induced EMT and glycolysis in EC cells through two distinct mechanisms: (i) DLEU2 induced HK2 expression by competitively binding with miR-455, and (ii) DLEU2 also interacted with EZH2 to silence a direct inhibitor of HK2, miR-181a.

**Conclusions:**

This study identified DLEU2 as an upstream activator of HK2-driven EMT and glycolysis in EC cells and provided significant mechanistic insights for the potential treatment of EC.

**Supplementary Information:**

The online version contains supplementary material available at 10.1186/s13046-021-02018-1.

## Background

Endometrial cancer (EC) is the most common gynecological cancer in developed countries, and its prevalence is rapidly increasing in Japan [1, 2]. Although many efforts have been made to develop targeted therapy and immunotherapy, the prognosis of patients with advanced-stage disease remains poor [3]. The mechanisms by which genetic and epigenetic alterations contribute to EC progression and influence the response to cancer therapies are incompletely understood.

Epithelial-to-mesenchymal transition (EMT) is a multistep process defined by the loss of epithelial phenotypes, and the gain of mesenchymal and cancer stem cell (CSC)-like characteristics [4, 5]. EMT can be triggered by various genetic and epigenetic signaling pathways in tumor cells [4]. Cancer cells frequently exhibit enhanced glycolysis and lactate production even in the presence of abundant oxygen, known as the Warburg effect or aerobic glycolysis [6]. Hexokinase 2 (HK2) is a glycolytic enzyme that catalyzes the first committed step in glucose metabolism, and its expression is markedly induced in cancer cells by multiple mechanisms [6]. Systemic HK2 deletion showed therapeutic effects in mice bearing lung tumors without adverse physiological consequences [7]. There is increasing evidence demonstrating an association between EMT execution and the reprogramming of glucose metabolism [8]. For instance, Snail serves as a positive regulator of EMT and glucose metabolism in gastric cancer [9], and HK2 was reported to increase glycolytic activity and ovarian cancer cell invasiveness through upregulation of the EMT activator, focal adhesion kinase (FAK) [10, 11], indicating that the overlapping mechanisms exist to affect both EMT and aerobic glycolysis.

Dysregulation of non-coding RNAs, including microRNAs (miRNAs) and long noncoding RNAs (lncRNAs), was known to be critical to cancer metastasis and metabolism [12–14]. LncRNAs mediate many important cancer phenotypes through their cross-talk with other macromolecules including DNA, RNA, and protein [15]. Although lncRNA DLEU2 is implicated in the tumorigenesis and progression of several malignancies [16, 17], the functional role and the mechanisms underlying its function in EMT and glycolysis in EC are still unknown.

In this study, we show that HK2 confers an oncogenic function in promoting EMT and glycolysis in EC cells. DLEU2 employs two distinct mechanisms to induce EMT and sustain glycolysis through HK2. First, DLEU2 competitively binds with miR-455 to induce HK2 expression. Second, DLEU2 interacts with EZH2 to silence a direct inhibitor of HK2, miR-181a.

## Materials and methods

### Human cell lines and culture conditions

We obtained HEC-1 and HEC-50 cells from JCRB Cell Bank (Osaka, Japan), Ishikawa and KLE cells from the American Type Culture Collection (Manassas, VA, USA), HHUA cells from RIKEN cell bank (Tsukuba, Japan), and the immortalized human endometrial epithelial cell line EM from Dr. Satoru Kyo (Shimane University, Japan). Sphere-forming derivatives and paclitaxel (TX)-resistant derivatives of HEC-50 cells were established as previously reported [18]. These cells were cultured in DMEM/F12 media (Sigma-Aldrich, St. Louis, MO, USA) supplemented with 10% fetal bovine serum (Invitrogen, Carlsbad, CA, USA).

### RNA extraction and qRT-PCR analysis

Total RNA was extracted using TRIzol reagent (Invitrogen). For mRNA analysis, RNA was reverse transcribed using a Reverse Transcription Kit (Takara, Japan). Real-time PCR was subsequently performed using Takara SYBR Premix Ex Taq II (Takara, Japan) on the ABI7300 PCR instrument. All primers (except DLEU2) were obtained from the PrimerBank database (http://pga.mgh.harvard.edu/primerbank/). The primers to assess DLEU2 expression have been previously reported [19] (forward: 5′-TCTGGAGAACAGCCTCACTTC-3′; reverse: 5′-TGCTGAGCTAAGTAGAGGTCTC-3′). All mRNA quantification was normalized to *GAPDH*. For miRNA detection, real-time PCR was conducted using the NCode miRNA qRT-PCR analysis (Invitrogen). The forward primers for miRNA analysis had the same sequences as the mature miRNAs. All miRNA data were normalized to *U6*.

### Supplemental information

Supplemental information (including supplemental experimental procedures and supplemental figures) for this article can be found online.

## Results

### HK2 is overexpressed in EC and predicts a worse prognosis

**Fig. 1:**
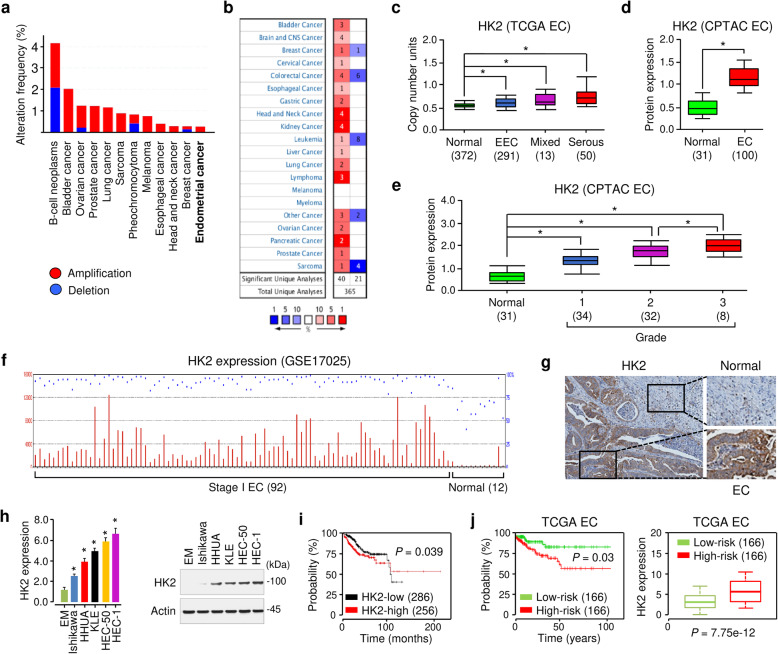
HK2 is overexpressed in EC and predicts a worse prognosis. **a** Genomic profiling of HK2 across human cancers was determined using cBioPortal. **b** Analysis of HK2 expression in different cancer tissues compared with corresponding normal tissues in datasets available at Oncomine. Red: upregulation; blue: downregulation. The numbers in the boxes represent the number of studies that met our thresholds. **c** DNA copy number profile of *HK2* in endometrial cancer (EC) tissues and normal tissues using the TCGA data from Oncomine. EEC: endometrial endometrioid carcinoma. **d** The protein expression of HK2 in EC and normal tissues was analyzed using UALCAN. **e** The protein expression of HK2 in normal tissues and subgroups of patients with EC stratified based on tumor grade (UALCAN). **f** The microarray dataset (GSE17025) was analyzed for HK2 expression in stage I EC samples and normal endometrium samples. **g** The protein expression of HK2 was examined in EC tissue and adjacent normal tissues. Images were downloaded from Human Protein Atlas. **h** *HK2* expression was measured in a normal endometrial cell line (EM) and human EC cell lines using quantitative real-time PCR and western blotting analysis. **i** Kaplan-Meier overall survival analysis was used to assess EC patients with high or low *HK2* expression from KM Plotter. **j** The patients from the TCGA EC dataset in SurvExpress were divided into low- and high-risk groups, and survival differences between the two groups were compared using Kaplan-Meier analysis (left panel). The expression level of HK2 in low- and high-risk groups was examined (right panel). *P < 0.05

The analysis of public datasets from The Cancer Genome Atlas (TCGA) (http://cancergenome.nih.gov) via the cBioPortal database (https://www.cbioportal.org/) showed that HK2 was frequently amplified in various human cancers, including EC (Fig. 1a). We compared the expression level of *HK2* in human tumors and normal tissues using the visualization tools provided by the Oncomine database (https://www.oncomine.org). *HK2* gene expression data were collected in a total of 365 different types of tumor studies (Fig. 1b). The default filter settings used were as follows: *p*-value of 1E-4, fold change of 2, and gene ranking of top 10%. As a result, 61 studies were statistically significant for *HK2* expression (40 studies for increased *HK2* expression, and 21 studies for reduced *HK2* expression (Fig. 1b). According to Oncomine, we found a significant increase in the DNA copy number of *HK2* gene in different subtypes of EC tissues (endometrioid, *p* = 4.43E-5; mixed, *p* = 0.018; serous, *p* = 8.22E-7) compared with normal tissues (Fig. 1c). We evaluated the correlation between aberrant HK2 expression and the clinicopathological features of EC patients in the Clinical Proteomic Tumor Analysis Consortium (CPTAC) datasets via the UALCAN database (http://ualcan.path.uab.edu/). The protein expression of HK2 was significantly higher in EC tissues compared to normal tissues (Fig. 1d), and its expression level was associated with a higher tumor grade (Fig. 1e).

The microarray gene expression dataset (GSE17025) from the GEO website was used to examine the expression of HK2 in EC tissues. HK2 was more highly expressed in stage 1 EC compared with normal endometrium (Fig. 1f). By analyzing the immunohistochemical staining images from the Human Protein Atlas database (https://www.proteinatlas.org/), we found that the protein expression of HK2 in EC tissues was dramatically higher than adjacent normal tissues (Fig. 1g).

The mRNA and protein expression of HK2 was higher in EC cells, compared with normal endometrial epithelial cells (Fig. 1h). Then, we assessed the prognostic value of *HK2* expression in EC using the KM Plotter database (http://kmplot.com/analysis/). The results showed higher mRNA expression of *HK2* was significantly correlated with the worse prognosis (Fig. 1i). When the EC tissues were divided into low- and high-risk groups for worse prognosis using the SurvExpress database (http://bioinformatica.mty.itesm.mx:8080/Biomatec/SurvivaX.jsp), higher *HK2* expression was evident in the high-risk group compared with the low-risk group (Fig. 1j).

### Inhibition of HK2 reverses the EMT phenotypes in EC cells

**Fig. 2:**
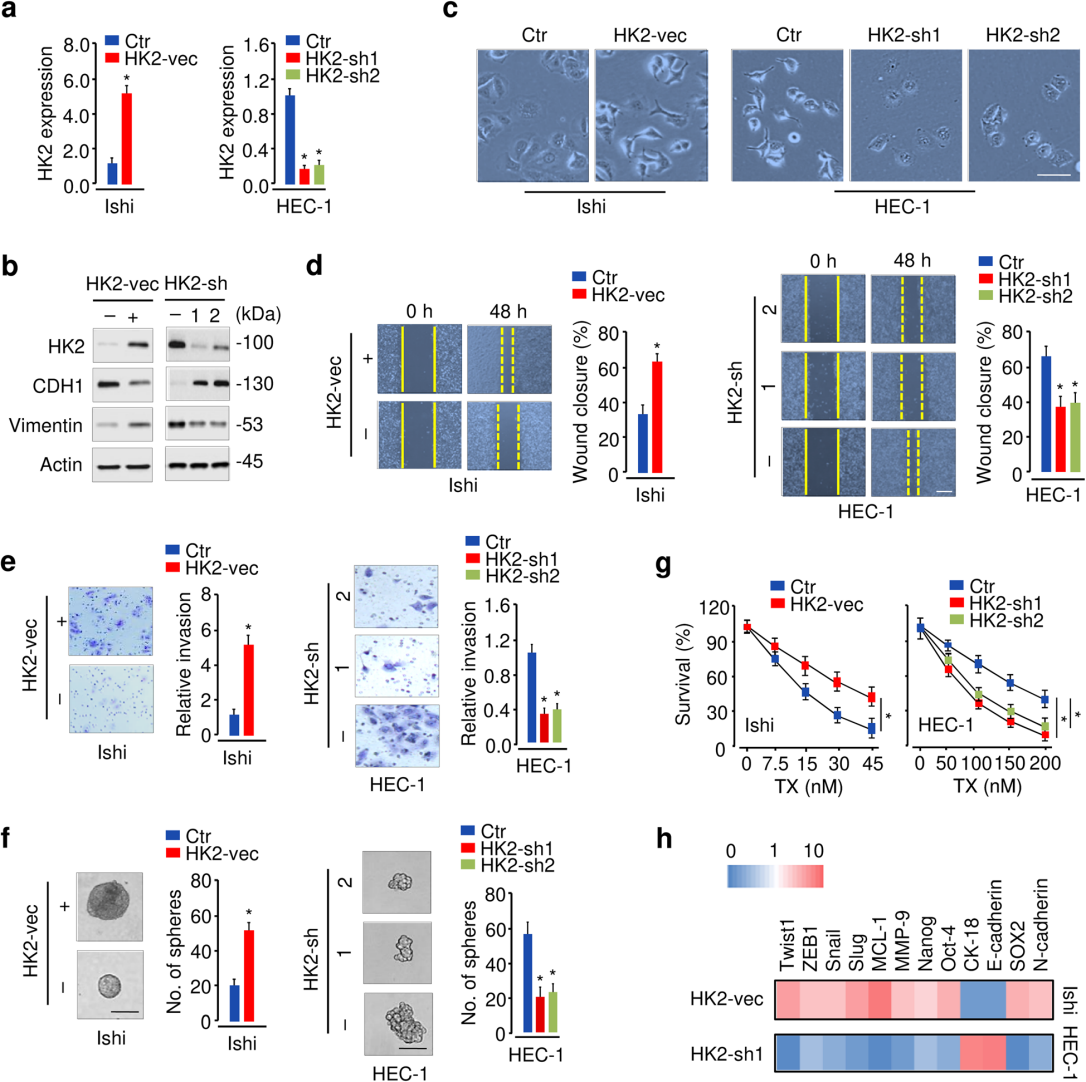
HK2 promotes the EMT phenotypes in EC cells. **a** qRT-PCR analysis of HK2 expression in EC cells after overexpression or knockdown of HK2. **b** Western blotting analysis of HK2, E-cadherin (CDH1), and Vimentin expression in EC cells transfected as indicated. **c** HK2 affects EC cell morphology. **d, e, f** Migration (**d**), invasion (**e**), and sphere formation (**f**) of EC cells after overexpression or knockdown of HK2. **g** Cell survival was examined by a cell viability assay in EC cells transfected as indicated and treated with TX. **h** A heat map of qRT-PCR results shows the expression of the indicated genes in EC cells upon overexpression or knockdown of HK2. Red: upregulation; blue: downregulation. Ishi: Ishikawa; vec: vector; sh: shRNA. *P < 0.05

Given that high HK2 expression was detected in EC, we assumed that HK2 could exert oncogenic functions in this cancer. We initially studied the effects of HK2 overexpression or knockdown on the proliferation of EC cells. Ishikawa (Ishi) cells that have relatively lower HK2 expression were stably transfected with an HK2 expression vector or a control vector (Fig. 2a, b). Alternatively, HEC-1 cells that exhibit relatively higher levels of HK2 were transfected with shRNAs specific for HK2 or a control shRNA (Fig. 2a, b). Indeed, overexpression of HK2 in Ishikawa cells caused a significant promotion of cell proliferation (Fig. S1). Consistent with this result, the downregulation of HK2 in HEC-1 cells significantly suppressed cell proliferation (Fig. S1).

Next, we examined the effect of HK2 expression on cell morphology, EMT, invasion, sphere formation, and drug resistance. HK2 overexpression led to a more spindle-like shape, and HK2 inhibition induced a more cobblestone-like appearance (Fig. 2c). In cell functional experiments, we found that the overexpression of HK2 in Ishikawa cells significantly increased the migration, invasion, sphere-forming ability and drug resistance to TX, whereas knocking down of HK2 in HEC-1 cells significantly decreased cell migration, invasion, sphere formation, and cellular resistance (Fig. 2d, e, f, g).

To understand the mechanisms associated with HK2-induced EMT, we examined the mRNA expression of EMT-related genes after overexpression or knockdown of HK2. Our results revealed that the expression of *CK-18* and *E-cadherin* were downregulated, and the expression of genes related to EMT induction and cancer aggressiveness, including *Twist*, *Snail*, *SOX2* and Vimentin, were upregulated upon HK2 overexpression (Fig. 2b, h). Conversely, the levels of E-cadherin and *CK-18* were induced, and those genes related to EMT induction and cancer aggressiveness were downregulated upon knockdown of HK2 (Fig. 2b, h). To explore the role of HK2 in vivo, we constructed mouse models using HEC-1 cells. The HK2-silenced group showed a significant reduction in tumor volume compared to the control groups (Fig. S2a, S2b). TX treatment alone showed slight effects in suppressing tumor growth (Fig. S2a, S2b). However, the combination of HK2 expression knockdown and TX treatment exhibited significant tumor growth suppression when compared to HK2 expression knockdown or TX treatment alone (Fig. S2a, S2b). Together, these results suggested that HK2 plays an oncogenic role in EC through mediating the EMT process.

### HK2 induces EMT and enhances glycolysis in EC by activating FAK and its downstream ERK1/2 signaling

**Fig. 3:**
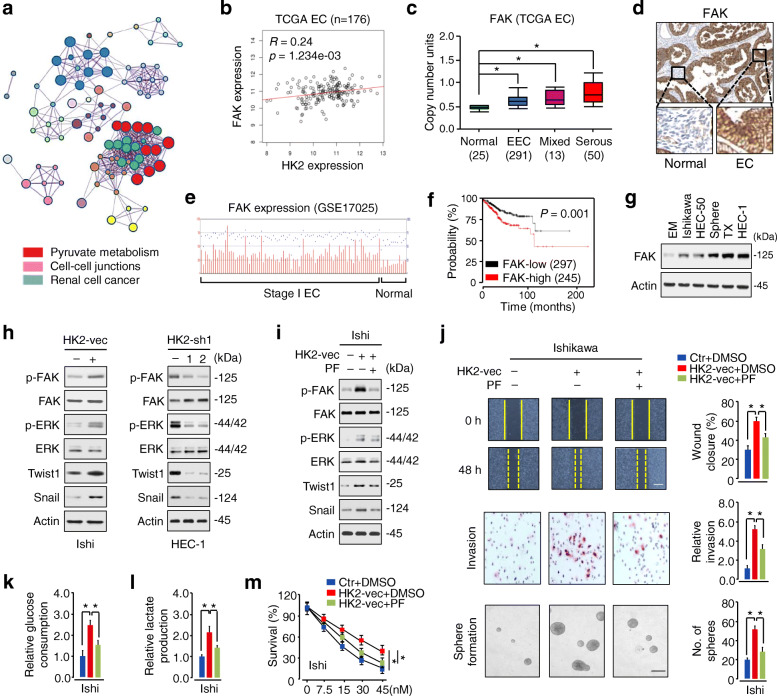
HK2 initiates EMT and enhances glucose metabolism in EC cells by activating the FAK/FAK/ERK1/2 pathway and upregulating Twist1 and Snail expression. **a** The top 100 genes that show a positive or negative co-expression correlation with HK2 in the TCGA EC dataset were selected and uploaded to Metascape for GO term detection and clustering. Same-colored dots fall into a function similar to the given title. Only the top 20 significant GO categories were shown. **b** Pearson correlation analysis revealed that *HK2* was significantly correlated with *FAK* in the TCGA EC dataset using LinkedOmics. **c** Analysis of *FAK* levels in EC and normal tissues using the TCGA data from UALCAN. **d** The protein expression of FAK in EC and adjacent normal tissues. Images were downloaded from Human Protein Atlas. **e** The microarray dataset (GSE17025) was analyzed for FAK expression in stage I EC samples and normal endometrium samples. **f** Kaplan-Meier curves show the overall survival of EC patients with high or low *FAK* expression from KM Plotter. **g** Western blotting analysis of FAK expression in a normal endometrial cell line (EM) and human EC cell lines, including Ishikawa, HEC-1, HEC-50, and sphere-forming (sphere) or TX-resistant (TX) HEC-50 derivatives. **h** Western blotting analysis of the indicated proteins in EC cells following overexpression or knockdown of HK2. **i** Western blotting analysis of the indicated proteins in Ishikawa cells expressing HK2, in the presence or absence of FAK inhibitor PF-573,228 (PF). **j**, **k**, **l** Cell migration, invasion, sphere formation, glucose consumption, and lactate production of Ishikawa cells expressing the control vector or HK2 vector, in the presence or absence of FAK inhibitor PF. **m** Ishikawa cells were transfected with or without the HK2 vector and treated with TX. Cell survival was examined by a cell viability assay in the presence or absence of PF. Ishi: Ishikawa; vec: vector; sh: shRNA. *P < 0.05

To investigate the signal pathways regulated by HK2 in EC, we analyzed series of genes that have strong co-expression correlation (Pearson r value > 0.2 or < − 0.2) with *HK2* in TCGA EC dataset via the LinkedOmics database (http://www.linkedomics.org), and selected the top 100 positively and 100 negatively correlated genes (Fig. S3). Then, we performed Gene Ontology (GO) and Kyoto Encyclopedia of Genes and Genomes (KEGG) functional enrichment analysis using the Metascape database (https://metascape.org/gp/index.html#/main/step1). Finally, the top 20 significant pathways and functions were selected according to *p* values, and the regulation of pyruvate metabolism, the regulation of cell-cell junctions, and renal cell cancer were significantly enriched biological processes (Fig. 3a), indicating that HK2-related signaling is potentially linked to EC metabolism and progression.

FAK, a known oncoprotein, is frequently overexpressed in human cancers and promotes cancer progression and metastasis [20]. Interestingly, we found that the expression of *FAK* was positively correlated with *HK2* expression in the TCGA EC dataset, as defined by the LinkedOmics database (Fig. 3b). This association led us to examine the expression of FAK in normal and EC tissues. By analyzing the TCGA data from the UALCAN database, we validated a significantly higher expression of *FAK* in EC tissues compared with normal tissues (Fig. 3c). The upregulation of FAK in EC tissues was further confirmed using the Human Protein Atlas database (Fig. 3d). Similarly, GSE17025 dataset in the GEO website showed significant overexpression of FAK in EC compared with normal tissues (Fig. 3e). Survival analysis of EC data via the KM Plotter database showed that high levels of *FAK* were associated with poor patient prognosis (Fig. 3f). Consistent with these data, western blotting analysis revealed that FAK was expressed at considerably higher levels in EC cell lines, compared with normal cells (Fig. 3g).

Since previous reports indicated that HK2-mediated ovarian cancer cell invasion is mediated through the FAK/ERK1/2 pathway [10], and FAK promotes EMT in cancer cells by upregulating Twist1 and Snail expression [21, 22], we examined whether HK2 regulates EMT in EC cells in a FAK-dependent manner. As expected, overexpression of HK2 induced FAK/ERK phosphorylation and the protein expression of Twist1/Snail in Ishikawa cells, whereas knockdown of HK2 expression with shRNAs reduced FAK/ERK phosphorylation and decreased the protein levels of Twist1/Snail in HEC-1 cells (Fig. 3h).

In addition, specific inhibition of FAK activity with a small molecule inhibitor PF-573,228 (PF) attenuated HK2-induced FAK/ERK phosphorylation and Twist1/Snail protein expression in Ishikawa cells (Fig. 3i). Moreover, treatment with PF also abrogated HK2-promoted cell migration, invasion, sphere formation, glucose consumption, lactate production and chemoresistance (Fig. 3j, k, l, m). Taken together, these results suggested that HK2 initiates EMT and enhances glucose metabolism in EC cells by activating FAK and its downstream signaling pathways (including ERK1/2, Twist1, and Snail).

### MiR-455 and miR-181a repress HK2 expression in EC cells

**Fig. 4:**
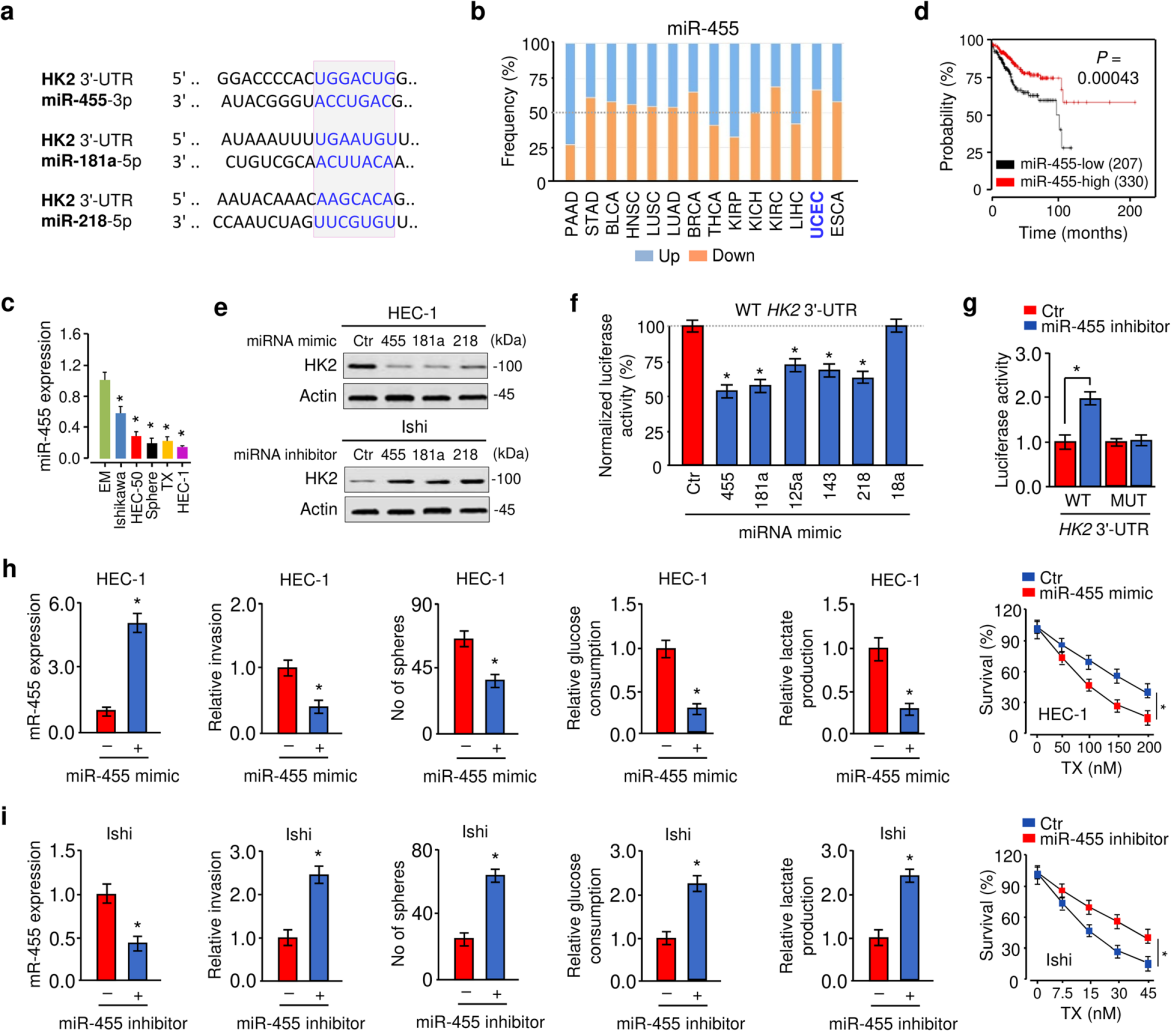
MiR-455 and miR-181a repress HK2 expression in EC cells. **a** The putative binding sites for miR-455, miR-181a and miR-218 in the *HK2* 3′-UTR. **b** Pan-cancer analysis of miR-455 expression in various human cancerous tissues, including endometrial (UCEC), relative to their paired normal tissues using BioExpress database. **c** qRT-PCR analysis of miR-455 expression in EM and EC cells. **d** Kaplan-Meier overall survival analysis was used to assess EC patients with high or low miR-455 expression based on the TCGA data with KM Plotter. **e** Western blotting analysis of HK2 expression in HEC-1 cells overexpressing indicated miRNA or Ishikawa cells with indicated miRNA expression knockdown. **f** Luciferase reporter assays of HEC-1 cells co-transfected with luciferase construct containing the wild-type (WT) *HK2* 3′-UTR, along with each indicated miRNA mimic or control (Ctr) mimic. **g** Luciferase reporter assay with Ishikawa cells co-transfected with a luciferase reporter plasmid containing WT or mutant (MUT) *HK2* 3′-UTR, along with miR-455 inhibitor or Ctr inhibitor. **h** Cell invasion, sphere formation, glucose consumption, and lactate production of HEC-1 cells transfected with Ctr mimic or miR-455 mimic. HEC-1 cells were transfected with or without miR-455 mimic and treated with TX. Cell survival was examined by a cell viability assay. **i** Cell invasion, sphere formation, glucose consumption, and lactate production of Ishikawa cells transfected with miR-455 inhibitor or Ctr inhibitor. Ishikawa cells were transfected with or without miR-455 inhibitor and treated with TX. Ishi: Ishikawa *P < 0.05

Next, we sought to understand how HK2 is upregulated in EC. Several prediction algorithms (TargetScan, microRNA.org, and DIANA-MicroT-CDS) were used to analyze the miRNAs targeting *HK2*. We noticed that three miRNAs (miR-455, miR-181a, and miR-218) potentially bind to the 3′-UTR of human *HK2* mRNA (Fig. 4a). MiR-181a and miR-218 possessed tumor suppressor activities in EC [23, 24]. MiR-455 is of particular interest, because its expression is significantly decreased in both endometrioid and serous EC tissues relative to normal endometrial tissues [25]. Consistent with this prior observation, our analysis of RNA sequencing data from the BioXpress database (https://hive.biochemistry.gwu.edu/bioxpress) showed the downregulation of miR-455 in EC tissues compared with normal tissues (Fig. 4b). We also found that EC cells exhibited downregulation of miR-455 and miR-181a when compared with normal cells (Fig. 4c; Fig. S4a). Moreover, the Kaplan-Meier plot of human EC demonstrated poorer survival with low expression of miR-455 or miR-181a (Fig. 4d; Fig. S4b). These data suggested the possibility that HK2 is negatively regulated by these miRNAs in EC cells.

To test this, we performed western bot analysis and found that ectopic expression of miR-455, miR-181a, and miR-218 decreased the protein level of HK2, whereas inhibition of miR-455, miR-181a, and miR-218 increased the protein expression of HK2 in EC cells (Fig. 4e). Our luciferase reporter assays showed that HEC-1 cells transfected with miR-455 mimic or miR-181a mimic showed a significant reduction of the luciferase activity of the wild-type *HK2* 3′-UTR, with respect to those cells transfected with control mimic (Fig. 4f; Fig. S4c). MiR-125a, miR-143, and miR-218 were used as positive controls (Fig. 4f), as HK2 has been previously shown to be a target of these miRNAs [26–28]. An unrelated miR-18a was used as a negative control (Fig. 4f). When the wild-type *HK2* 3′-UTR was co-transfected into Ishikawa cells with miR-455 inhibitor or miR-181a inhibitor, the luciferase activity of the wild-type *HK2* 3′-UTR was significantly induced (Fig. 4g; Fig. S4d). However, mutation of the binding site of miR-455 or miR-181a in the *HK2* 3′-UTR resulted in a recovery of luciferase activity (Fig. 4g; Fig. S4c, S4d). These data supported that miR-455 directly suppresses HK2 expression and promoted us to hypothesize that miR-455 could inhibit EC tumorigenesis and development by targeting HK2.

We next assessed whether miR-455 suppresses the malignant phenotypes of EC cells. Our qRT-PCR assays and cell functional assays showed that transfection with miR-455 mimic resulted in significant repression of cell invasion, sphere formation, chemoresistance, glucose consumption, and lactate production (Fig. 4h). Increased cell invasion, sphere formation, chemoresistance, glucose consumption, and lactate production was confirmed in miR-455 inhibitor-transfected cells compared with control inhibitor-transfected cells (Fig. 4i). Consistent with these data, transfection with miR-455 mimic reduced the activity of FAK and ERK and Vimentin protein expression, while induced E-cadherin protein expression (Fig. S5). In contrast, inhibition of miR-145 by miR-455 inhibitor yielded an opposite effect on these downstream effectors of HK2 in Ishikawa cells (Fig. S5).

### DLEU2 enhances HK2 expression via competitively binding with miR-455

**Fig. 5:**
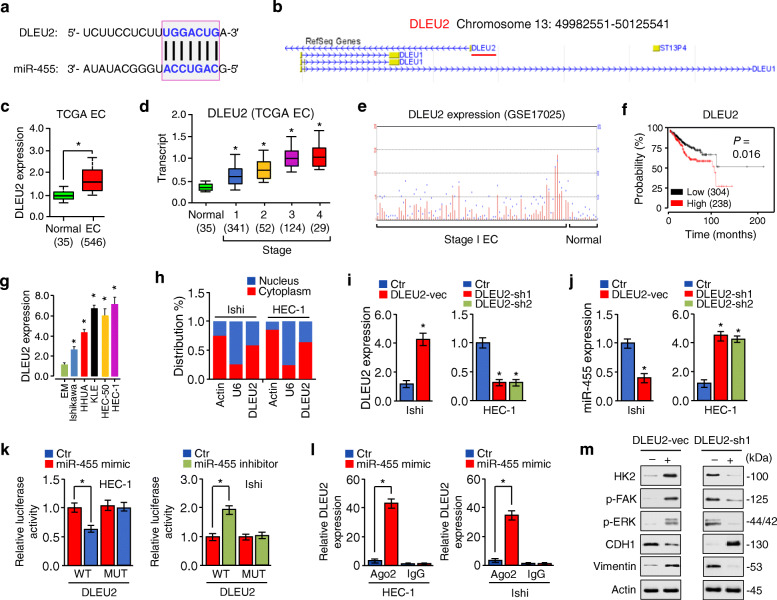
DLEU2 enhances HK2 expression via competitively binding with miR-455. **a** The miR-455 binding sequence in the DLEU2 sequence. **b** A schematic diagram shows that the *DLEU2* gene is located on chromosome 13q14.2 (red bar). **c** Analysis of DLEU2 expression in EC and normal tissues using the TCGA data from UALCAN. **d** The expression of DLEU2 in normal tissues, and subgroups of patients with EC stratified based on tumor stage (UALCAN). **e** The NCBI Gene Expression Omnibus (GEO) dataset GSE17025 was used for profiling DLEU2 expression in stage I EC samples and normal endometrium samples. **f** Kaplan-Meier overall survival analysis was used to assess EC patients with high or low DLEU2 expression based on the TCGA data from KM Plotter. **g** qRT-PCR analysis of DLEU2 expression in the normal endometrial epithelial cell line EM and EC cell lines. **h** qRT-PCR analysis of the relative DLEU2 expression after nuclear and cytoplasmic RNA separation. *GAPDH* was used as a cytoplasmic marker, and *U6* was used as a nuclear marker. **i, j** qRT-PCR analysis of DLEU2 (**i**) and miR-455 (**j**) expression upon overexpression or knockdown of DLEU2 in EC cells. **k** Luciferase reporter assay with EC cells co-transfected with a luciferase reporter plasmid containing the wild-type (WT) or mutant (MUT) DLEU2, together with miR-455 mimic, miR-455 inhibitor, or their respective controls. **l** RIP assays were performed in EC cells that transiently overexpressed miR-455 mimic or control mimic, followed by qRT-PCR analysis to detect DLEU2 expression associated with Ago2. **m** Western blotting analysis of the indicated proteins in EC cells upon overexpression or knockdown of DLEU2. Ishi: Ishikawa; vec: vector; sh: shRNA. *P < 0.05

In addition to functioning at the transcriptional level in the nucleus, lncRNAs can regulate gene expression by acting as competing RNAs for specific miRNAs in the cytoplasm [15]. To investigate whether lncRNAs can regulate HK2 expression through miR-455, we predicted those lncRNAs with the potential to interact with miR-455 using the bioinformatics tool starBase v3.0 (http://starbase.sysu.edu.cn), and identified DLEU2 as a potential candidate lncRNA (Fig. 5a). According to data from the Atlas of Genetics and Cytogenetics in Oncology and Haematology database (http://atlasgeneticsoncology.org/) and lncATLAS database (https://lncatlas.crg.eu/), DLEU2 is located on chromosome 13q14.2 and is present in the cytoplasm and nucleus of human cancer cell lines, including A549, HeLa, and K562 cells. (Fig. 5b; Fig. S6a). Analysis of the UALCAN and GENT database (http://gent2.appex.kr/gent2/) showed that DLEU2 overexpression occurs in various cancer types (including EC) (Fig. 5c; Fig. S6b). Interestingly, the expression of DLEU2 was gradually increased from stage 1 to stage 3 (Fig. 5d). Using the human microarray dataset (GSE17025), we found that DLEU2 was expressed at higher levels in stage 1 EC tissues, but hardly detectable in normal endometrial tissues (Fig. 5e). Survival analysis suggested that high DLEU2 expression was significantly correlated with poor overall survival (Fig. 5f). The relationship between overexpression of DLEU2 and worse patient survival rate was also confirmed in esophageal, head-neck, kidney, liver cancers, and pheochromocytoma/paraganglioma (Fig. S7).

The endogenous expression of DLEU2 was examined in EC cell lines and normal cells, and the qRT-PCR results revealed that it was significantly upregulated in EC cells (Fig. 5g). Through the nuclear-cytoplasmic fractionation assay, we found that DLEU2 was mainly distributed in the cytoplasm of EC cells (Fig. 5h). Then, we investigated the effects of either overexpression or knockdown of DLEU2 on the levels of miR-455 using qRT-PCR assays, and observed that the overexpression of DLEU2 decreased, whereas the suppression of DLEU2 increased miR-455 expression (Fig. 5i, j).

To identify the mechanism by which DLEU2 represses miR-455 expression, we co-transfected EC cells with luciferase reporter vectors containing wild-type or mutant DLEU2, together with (or without) miR-455 mimic or miR-455 inhibitor. The dual-luciferase assays showed that miR-455 directly bond to DLEU2 in EC cells (Fig. 5k). The RIP assay was conducted using EC cells that transiently overexpressed miR-455 and showed that the endogenous DLEU2 was significantly enriched in EC cells transfected with miR-455 mimic than in the control cells transfected with control mimic (Fig. 5l). In addition, overexpression of DLEU2 in Ishikawa cells increased the protein expression of HK2 and Vimentin as well as the phosphorylation of FAK and ERK, but reduced the levels of E-cadherin (Fig. 5m). DLEU2 knockdown had opposite effects on these aberrations (Fig. 5m), suggesting that DLEU2 induces HK2 expression via competitively binding with miR-455.

### Aberrant expression of DLEU2 drives EMT and glycolysis in EC

**Fig. 6:**
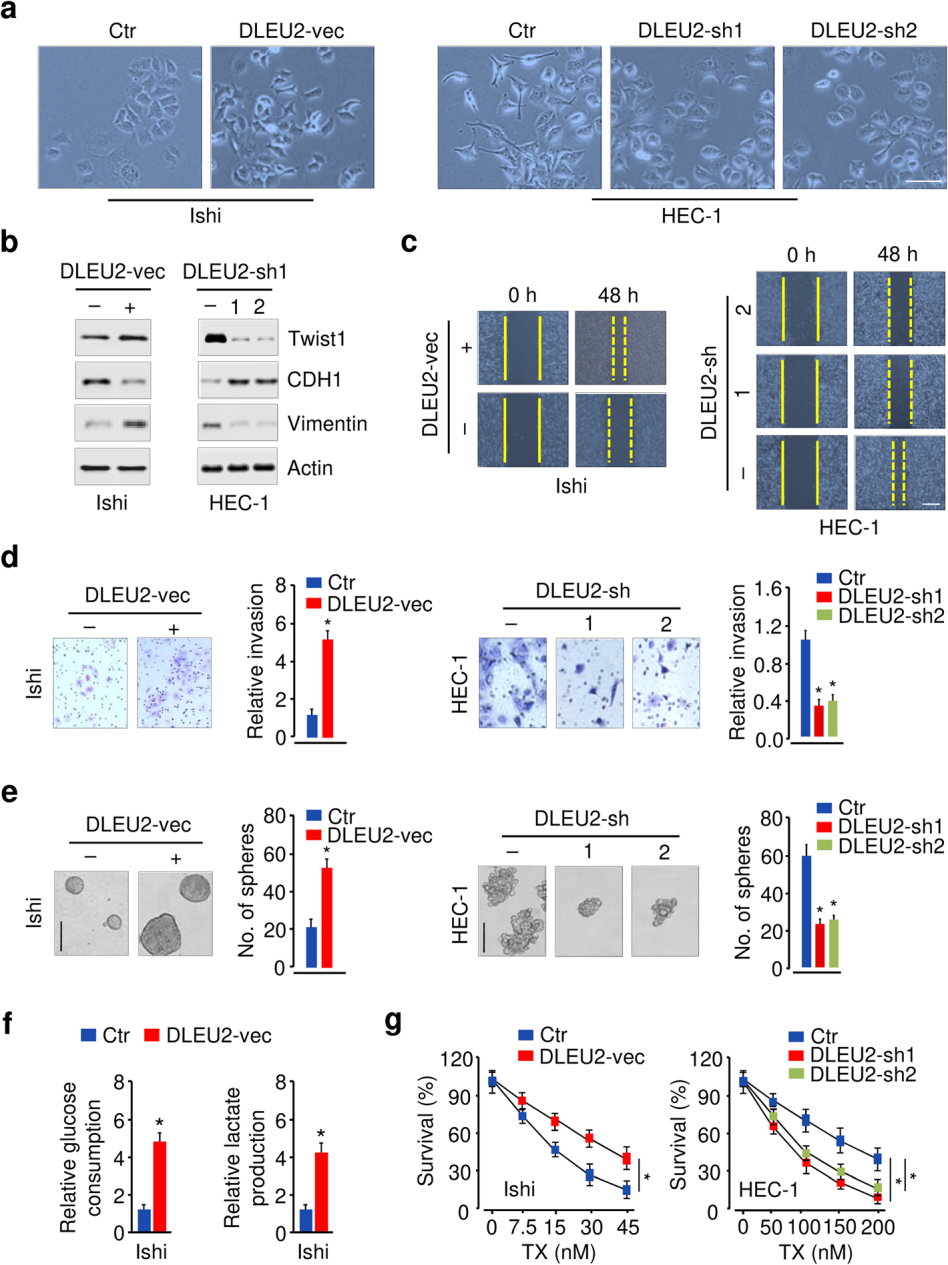
Aberrant expression of DLEU2 drives EMT and glycolysis in EC. **a** Morphological changes upon functional overexpression or inhibition of DLEU2. **b** Western blotting analysis of the indicated proteins in EC cells following overexpression or knockdown of DLEU2. **c**, **d**, **e** Cell migration (**c**), invasion (**d**), sphere formation (**e**) of EC cells upon overexpression or inhibition of DLEU2. **f** Cell glucose consumption and lactate production of Ishikawa cells following overexpression of HK2. **g** Cell viability assay to assess the effects of DLEU2 knockdown on the cellular response to TX. Ishi: Ishikawa; vec: vector; sh: shRNA. *P < 0.05

We investigated if DLEU2 is required for the EMT characteristics and glycolysis in EC cells. Ectopic expression of DLEU2 led to a fibroblast-like mesenchymal appearance of cells with a loss of epithelial features consistent with EMT (Fig. 6a). These morphological changes were consistent with defined molecular alterations that occur during the induction of EMT, as demonstrated by decreased expression of epithelial marker E-cadherin and increased expression of mesenchymal markers, vimentin and Twist1 (Fig. 6b). In contrast, silencing of DLEU2 resulted in changes in morphology and gene expression consistent with a mesenchymal-to-epithelial transition, where the cells reverted from a more spindle-like morphology to an epithelial-like phenotype (Fig. 6a, b). Cell functional assessment showed that DLEU2 overexpression significantly induced, and DLEU2 inhibition consistently reduced cell migration, invasion, sphere formation, glucose consumption, lactate production, and chemoresistance (Fig. 6c, d, e, f, g; Fig. S8). Furthermore, we evaluated the therapeutic value of DLEU2 inhibition on EC in vivo. Knockdown of DLEU2 significantly inhibited EC growth (Fig. S9). The mice receiving combined treatment of DLEU2 expression knockdown and TX treatment demonstrated a much smaller tumor volume and weight than the other mice (Fig. S9). Overall, these data suggested that DLEU2 controls glycolysis and is necessary for the maintenance of mesenchymal phenotypes and drug-resistance capacity of EC cells.

### DLEU2 binds to EZH2 protein, resulting in decreased miR-181a levels

**Fig. 7:**
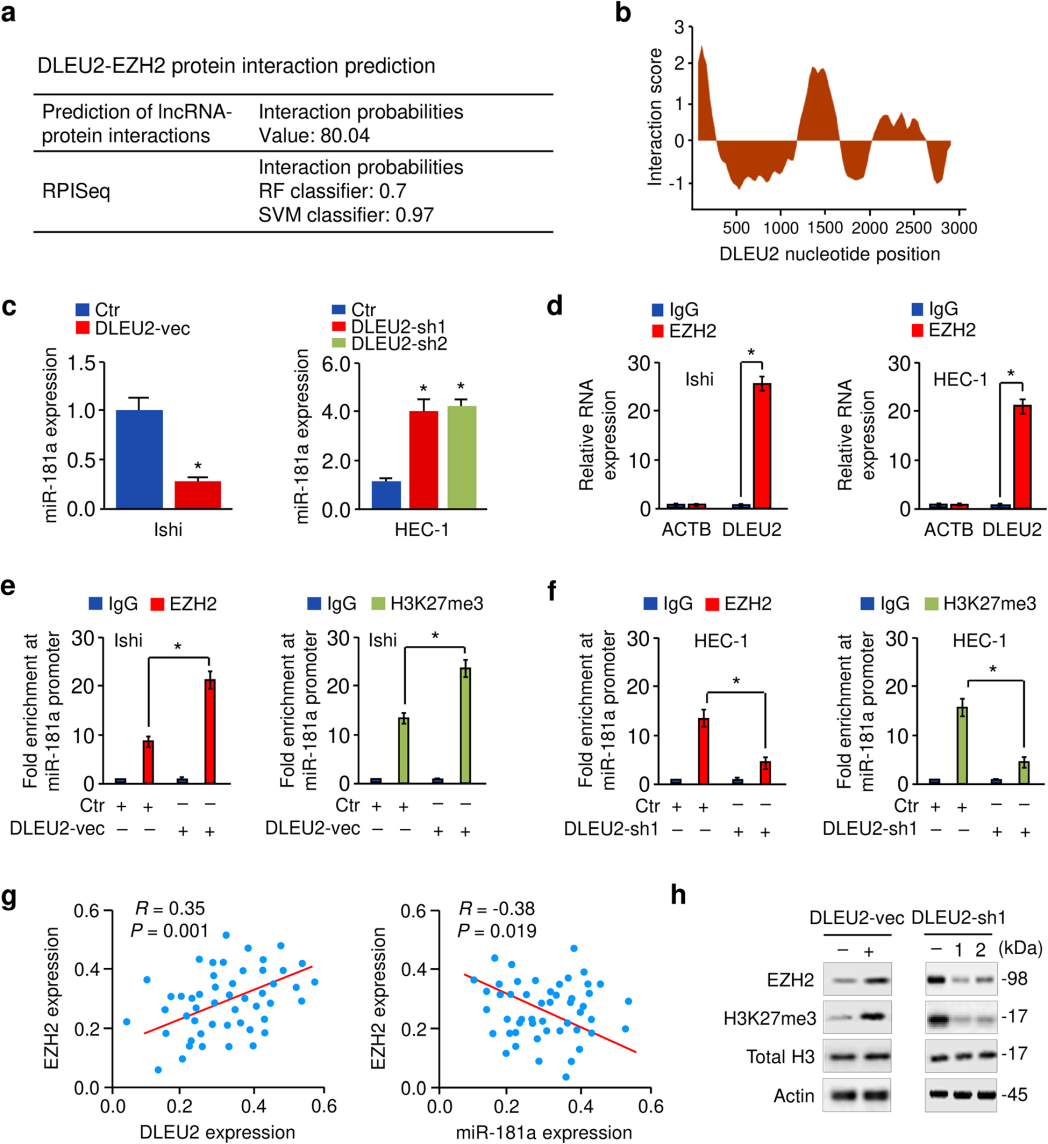
DLEU2 binds to EZH2 protein, resulting in decreased miR-181a levels. **a** Bioinformatics software (Prediction of lncRNA-protein interactions and RPISeq) predicted that there was a strong interaction propensity between DLEU2 and EZH2. In the prediction of the lncRNA-protein interactions database, interaction probabilities > 50 indicated that a strong interaction probability between the corresponding RNA and protein. In RPISeq, predictions with probabilities > 0.5 indicated that the corresponding protein and RNA are likely to interact. **b** Prediction of interaction propensity between DLEU2 and EZH2 (catRAPID). A positive interaction score predicts an increased propensity for binding. **c** qRT-PCR analysis of miR-181a expression following overexpression or knockdown of DLEU2. **d** RIP assays show that DLEU2 bound to EZH2 in EC cells. *ACTB* mRNA was used as the negative control. **e, f** The effects of either DLEU2 overexpression (**e**) or DLEU2 knockdown (**f**) on the binding of EZH2 and H3K27me3 to the miR-181a promoter were examined using ChIP-qPCR assays. **g** The correlation between DLEU2, EZH2, and miR-181a expression was detected in EC patients. Ishi: Ishikawa; vec: vector; sh: shRNA. **h** Western blotting analysis of the indicated proteins in EC cells upon overexpression or knockdown of DLEU2. *P < 0.05

Several lncRNAs, such as DLEU2, influence the expression of protein-coding genes by interacting with the PRC2 subunit EZH2 [17]. We predicted the interaction probabilities of DLEU2 and EZH2 via the Prediction of lncRNA-protein interactions (http://bioinfo.bjmu.edu.cn/lncpro/) and RPISeq databases (http://pridb.gdcb.iastate.edu/RPISeq/). We found that DLEU2 could bind to EZH2 (Fig. 7a). These results were further supported by catRAPID predictions (Fig. 7b), which estimates the binding potential through the secondary structure, hydrogen bonding, and van der Waals of both protein and RNA sequences, allowing the identification of binding partners with high confidence (http://s.tartaglialab.com/page/catrapid_omics_group).

Previous studies have shown that EZH2 regulates a wide variety of miRNAs (including miR-181a) by increasing the levels of the epigenetic silencing marker H3K27me3 [29], and EZH2 epigenetically silences tumor suppressor miRNAs through H3K27 trimethylation in EC cells [30]. Interestingly, miR-181a level was verified to be downregulated in EZH2-expressing cells as compared to the control cells, and knockdown of EZH2 resulted in increased miR-181a expression (Fig. S10a, S10b). Therefore, we investigated whether DLEU2 represses the expression of miR-181a via an EZH2-mediated mechanism. Our qRT-PCR analysis suggested that DLEU2 overexpression and knockdown significantly decreased and increased miR-181a levels, respectively (Fig. 7c). RIP assays also revealed that DLEU2 was significantly enriched with the EZH2 antibody compared with IgG (Fig. 7d). Two known EZH2-interactor lncRNAs (NEAT1 and HOTAIR) served as positive controls [31, 32] (Fig. S11), and *ACTB* was used as the negative control (Fig. 7d). To explore how DLEU2 affects miR-181a expression through EZH2, we performed ChIP-qPCR assays. Overexpression of DLEU2 significantly increased the binding ability of EZH2 and H3K27me3 to the promoter region of miR-181a, whereas DLEU2 depletion had the opposite effects (Fig. 7e, f). Furthermore, we found a positive correlation between DLEU2 and EZH2 expression and a negative correlation between EZH2 and miR-181a expression in EC tissues (Fig. 7g).

We noticed that the protein expression of EZH2 was elevated following overexpression of DLEU2 (Fig. 7h). In contrast, depletion of DLEU2 reduced EZH2 expression (Fig. 7h), indicating that DLEU2 is essential for the regulation of EZH2. Considering the role of DLEU2 in regulating the expression of miRNA, we hypothesized that DLEU2 may regulate the expression of EZH2 via some other miRNAs. Using starBase, we could detect the potential binding site between miR-582 and the 3′-UTR of *EZH2* mRNA, and between miR-582 and DLEU2 (Fig. S12a). MiR-582 exhibited significantly lower abundance in EC cells compared with normal cells (Fig. S12b). After overexpressing DLEU2 in Ishikawa cells, miR-582 expression was decreased (Fig. S12c). Moreover, the suppression of DLEU2 in HEC-1 cells significantly enhanced miR-582 expression (Fig. S12c). To further confirm whether miR-582 mediates EZH2 expression, we manipulated the level of miR-582 either through knockdown or overexpression (Fig. S12d). We found that the protein expression of EZH2 was inhibited by miR-582 in EC cells (Fig. S12e), suggesting the possibility that DLEU2 can enhance EZH2 expression through miR-582.

Together, these results demonstrated that DLEU2 upregulates EZH2 levels (probably via miR-582) and interacts with EZH2 to epigenetically silence miR-181a, consequently leading to increased HK2 expression.

### Correlated expression of DLEU2 and its downstream signaling molecules in EC

**Fig. 8:**
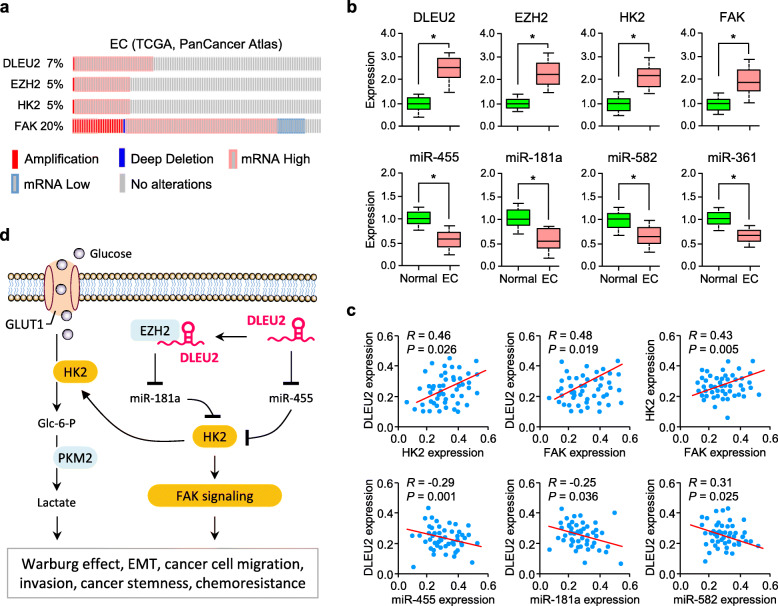
Correlated expression of DLEU2 and its downstream signaling molecules in EC. **a** Genomic profiling of DLEU2 and its downstream signaling molecules in human TCGA EC tissues determined by cBioPortal analysis. **b** qRT-PCR analysis of the indicated genes and miRNAs in EC and adjacent normal tissues. **c** Correlation of DLEU2 levels with the expression of its downstream signaling molecules in EC tissues as assessed using qRT-PCR analysis. **d** A scheme depicting how DLEU2 induces EMT and sustains glycolysis in EC via activating the HK2 signaling by competitively binding with miR-455 and by modulating the EZH2/miR-181a pathway. *P < 0.05

To understand the clinical relevance of the above findings, we examined DLEU2, *EZH2*, *HK2* and *FAK* expression in 507 human EC tissues (TCGA, PanCancer Atlas) downloaded from the cBioPortal database. We found that these genes possessed the concordant changes at both genomic DNA and mRNA levels, mainly gene amplification and mRNA upregulation (Fig. 8a).

In accordance with our data shown above, we confirmed the significant increase in DLEU2, *EZH2*, *HK2*, and *FAK* levels, and a significant decrease in miR-361, miR-455, miR-181a, and miR-582 levels in EC tissues compared with normal tissues, according to the results from qRT-PCR analysis (Fig. 8b). Expectedly, there was a significant positive correlation between the expression of DLEU2 and its downstream molecules (*HK2* and *FAK*) and between the levels of *HK2* and *FAK* (Fig. 8c), based on our experimental qRT-PCR validation studies. On the other hand, in EC tissues, DLEU2 drew a negative correlation with miR-455, miR-181a, and miR-582 (Fig. 8c). Overall, these results support our in vitro and in vivo findings and suggest the clinical relevance between DLEU2 and its downstream effectors in human EC.

## Discussion

Despite a better understanding of EC genetic and epigenetic changes in recent years, the development of metastatic dissemination and drug resistance remain the major challenges for treating this cancer. Previous evidence has indicated that EMT-guided invasive growth and enhanced aerobic glycolysis are closely connected with each other, and serve as two main manifestations of tumor progression [8–11]. Thus, unraveling the precise mechanisms that underlie both EMT and glycolysis would facilitate a deeper understanding of the biological complexity of cancer metastasis and may lead to more effective and selective cancer treatments.

Here, we describe a previously unappreciated regulatory mechanism whereby DLEU2, HK2, and FAK form a functional axis that plays a causal role in EMT and glycolysis, representing an advance in the field of genetic and epigenetic regulation of these features of EC cells. Importantly, we demonstrate that DLEU2 regulates the HK2/FAK/ERK1/2 signaling via competitively binding with miR-455 and by interacting with EZH2 to silence miR-181a expression (Fig. 8d).

DLEU2, HK2 and FAK are selectively overexpressed in EC cells but not in normal cells, act as key EMT/glycolysis-stimulating factors in EC, and are inversely correlated with patient prognosis. We also utilized EC cell models to illustrate that inhibition of DLEU2 and HK2 reduces cell migration, invasion, sphere formation and glycolysis, while enhancing chemotherapy sensitivities. These findings raise the possibility that targeting the DLEU2-driven HK2 signaling axis may provide a therapeutic strategy for EC patients with highly aggressive and glycolytic tumors.

Numerous studies have reported that aberrant HK2 overexpression stimulates cancer development, metastasis, and therapeutic resistance to chemotherapy and radiation [33–35]. HK2 is highly expressed in a wide range of human cancers [6] and is associated with poor outcomes of patients with diverse cancers [36]. Of note, forced overexpression of HK2 induces cancer cell invasiveness, EMT, and cancer stemness [37, 38]. Our present study has provided a better understanding of how enhanced HK2 expression confers aggressive tumor progression and poor prognosis in EC patients through the maintenance of mesenchymal state and increased glycolytic activity.

FAK promotes cell survival, growth, angiogenesis, migration, invasion, EMT, and CSC-like characteristics, thus controlling cancer initiation, progression, metastasis, recurrence, and drug resistance [20]. We found that the expression of FAK was significantly higher in EC tissues than in normal tissues. This was consistent with a previous study showing that FAK protein expression was increased in endometrial hyperplasia and EC tissues compared with normal endometrium, and FAK overexpression in EC correlates with higher FIGO grade [39]. Furthermore, our data suggest that the oncogenic function of HK2 in EC is due to its ability to activate FAK-dependent signaling, however, the precise mechanism by which this HK2/FAK axis works in EC remains largely elusive.

Previous evidence has indicated that FAK exerts its oncogenic functions through the modulation of multiple downstream signaling cascades, such as ERK1/2, PI3K/AKT, and JNK pathways [40]. Consistent with these findings, our results support that HK2 drives EMT and glycolysis through activation of FAK and its downstream ERK1/2 signaling. However, other researchers have found that FAK might trigger PI3K/AKT (but not ERK1/2) signaling to affect melanoma cell invasion and metastasis [41]. Importantly, in EC cells, treatment with the selective FAK inhibitor abolished estrogen-induced cell migration, and pharmacological inhibition of PI3K/AKT and ERK1/2 pathways prevented the phosphorylation of FAK [42], implying an interesting possibility whereby FAK might induce the activation of PI3K/AKT and/or ERK1/2 pathways, and then high PI3K/AKT and/or ERK1/2 activity feeds back to initiate FAK, thus establishing a positive feedback loop to mediate HK2-induced EMT and glycolysis in EC cells. Further studies are thus required to elucidate the detailed molecular regulatory circuitry between them, and to expand our understanding of the complexity of HK2-dependent gene networks contributing to the metastatic and metabolic phenotypes of EC cells.

MiRNAs are key regulators of the human transcriptome and dysregulation of miRNAs has a critical role in cancer metastasis and metabolism [12, 13]. Reduced expression of miR-455 and its tumor suppressor function has been reported in colon [43], pancreatic [44], lung [45], and prostate [46] cancers. MiR-455 inhibits the EMT process in pancreatic cancer [44] and hepatocellular carcinoma [47] by modulating the Wnt/β-catenin and STK17B/AKT/GSK-3β/Snail signaling, respectively. Here, we demonstrated a new function of miR-455 in suppressing EMT and glycolysis of EC cells via repressing the expression of HK2. Thus, these previous results, together with our findings, collectively suggest that miR-455 could suppress multiple oncogenic pathways, making it a novel therapeutic target for EC and other cancers. Future studies will reveal how miR-455 functions and loss of miR-455 expression occurs in human tumors.

The list of lncRNAs involved in tumor progression is expanding rapidly. Although a few lncRNAs have been implicated in EMT and cancer metabolism regulation [15, 48], the functions and underlying mechanisms of most lncRNAs in EC remain poorly understood. Previous studies on DLEU2 have mainly focused on its oncogenic role in controlling tumor cell proliferation, migration, and invasion [16, 17]. In this study, we found that the overexpression of DLEU2 could regulate HK2/FAK/ERK1/2 signaling through binding miR-455 and an EZH2/miR-181a axis-mediated mechanism, resulting in the gain of EMT and glycolytic properties in EC cells. Additional studies will be needed to clarify the functional contribution of DLEU2 in cancer biology, the precise mechanisms downstream of DLEU2, and how its expression is regulated in tumor cells.

Identifying potential biomarkers to detect EC in the early stage would contribute to improved survival rates. Although prior studies using genomic and proteomic technologies have provided molecular insights into the pathogenesis and biology of EC, but have yet to yield reliable biomarkers to impact the early diagnosis of this disease [49]. Overexpression of EZH2 has been previously discovered in pre-cancerous lesions of the endometrium and EC tissues, but not in normal endometrium samples [50]. On the basis of GEO EC datasets, we observed that EZH2 (data not shown), HK2, FAK, and DLEU2 exhibited significantly increased expression in stage I EC tissues compared to normal tissues (Figs. 1, 3, 5). Consistently, in the TCGA dataset, we verified their overexpression or amplification in EC tissues (Fig. 8). These results demonstrate that these molecules might be valuable biomarkers for the early diagnosis of EC.

## Conclusions

In summary, we demonstrate that a novel function of lncRNA DLEU2 in EC is to promote the acquisition of mesenchymal and glycolytic features by a mechanism that involves its ability to epigenetically increase HK2 expression, which drives EMT and glycolysis through activation of FAK. This study facilitates our understanding of the molecular basis responsible for the aggressive and glycolytic nature of human EC and offers significant mechanistic value for the potential treatment of EC.

## Supplementary Information

**Additional file 1.**

## Data Availability

The public datasets analyzed during the current study are available in the repositories listed below: • Gene Expression Omnibus: GSE17025 https://www.ncbi.nlm.nih.gov/geo/query/acc.cgi?acc=GSE17025 • The Cancer Genome Atlas: http://cancergenome.nih.gov/

## Abbreviations

EMT: Epithelial-to-mesenchymal transition
LncRNA: Long non-coding RNA
miRNA: MicroRNA
CSC: Cancer stem cell
