# Supplementary material for: Long non-coding RNA DLEU2 drives EMT and glycolysis in endometrial cancer through HK2 by competitively binding with miR-455 and by modulating the EZH2/miR-181a pathway
Source: J Exp Clin Cancer Res. 2021 Jun 26;40:216. doi: 10.1186/s13046-021-02018-1 (PMC8235565; doi:10.1186/s13046-021-02018-1)
Supplement: Supplementary file 1 — Additional file 1. [file 13046_2021_2018_MOESM1_ESM.docx]

Revised-Supplementary information

Long non-coding RNA DLEU2 drives EMT and glycolysis in endometrial cancer through HK2 by competitively binding with miR-455 and by modulating the EZH2/miR-181a pathway

Peixin Dong^1, †,^ *, Ying Xiong^2, †^, Yosuke Konno^1,^*, Kei Ihira^1^, Noriko Kobayashi^1^, Junming Yue^3, 4^ and Hidemichi Watari^1^

1. Department of Obstetrics and Gynecology, Hokkaido University School of Medicine, Hokkaido University, Sapporo, Japan
2. Department of Gynecology, State Key Laboratory of Oncology in South China, Sun Yat-sen University Cancer Center, Guangzhou, China
3. Department of Pathology and Laboratory Medicine, University of Tennessee Health Science Center, Memphis, TN 38163, USA
4. Center for Cancer Research, University of Tennessee Health Science Center, Memphis, TN 38163, USA

^†^ Contributed equally

* Correspondence: [dpx1cn@gmail.com](mailto:dpx1cn@gmail.com); konsuke013@gmail.com

**Supplemental materials and methods**

**Western blotting analysis**

Whole-cell protein extracts were obtained using M-Per Mammalian Protein Extraction Reagent (Pierce, Rockford, IL, USA). Equal amounts of protein samples were separated on 10% SDS-PAGE gels and transferred to nitrocellulose membranes. Membranes were blocked with 10% non-fat milk and probed by the following primary antibodies against HK2 (#2106; Cell Signaling, MA, USA), β-actin (#4967; Cell Signaling), E-cadherin (A01589; GenScript, NJ, USA), Vimentin (A01189; GenScript), phospho-FAK (44-624G; Invitrogen), FAK (sc-558; Santa Cruz Biotechnology, TX, USA), phospho-ERK (sc-7383; Santa Cruz Biotechnology), ERK (sc-514302; Santa Cruz Biotechnology), Twist (sc-81417; Santa Cruz Biotechnology), Snail (sc-10432; Santa Cruz Biotechnology), EZH2 (#5246; Cell Signaling), H3K27me3 (#9733; Cell Signaling) and Histone H3 (#9715; Cell Signaling) at 4°C overnight. After incubation with the corresponding secondary antibodies, membranes were exposed using the ECL detection kit (Amersham Pharmacia Biotech, UK). Primary and secondary antibodies were used at 1:1000 and 1:5000 dilutions, respectively.

**Generation of stable cell lines**

The pCMV6-HK2 construct containing human *HK2* cDNA and pCMV6 (control vector) were purchased from OriGene (Rockville, MD, USA). Plasmids encoding two short hairpin RNAs (shRNAs) targeting *HK2* (HK2-shRNAs) and a control shRNA were obtained from Santa Cruz Biotechnology. A plasmid pCMV-DLEU2 (custom synthesized by OriGene) was utilized to generate DLEU2-overexpressing cell lines. The above plasmids were then transfected into EC cells, and the selection of transduced cells was accomplished using G418 (Sigma-Aldrich, St. Louis, MO, USA) for 4 weeks. Two shRNAs that target lncRNA DLEU2 transcription (constructed by GenePharma, Shanghai, China; DLEU2-shRNAs) and a control shRNA (GenePharma) were transfected into EC cells and selected with puromycin (Sigma-Aldrich, St. Louis, MO, USA) for 4 weeks.

**Cell transfection**

For cell transfection, expression plasmids, siRNAs (Ambion, Austin, TX, USA), miRNA mimics (Ambion), and miRNA inhibitors (Ambion) were transfected using Lipofectamine 2000 (Invitrogen) according to the manufacturer’s instructions.

**Cell functional assays**

Cell proliferation, survival, migration, invasion, and sphere formation were investigated by Cell Counting Kit-8, cell viability, wound-healing, Matrigel invasion, and sphere formation assays, respectively, as previously described [1].

***In vivo* mouse experiments**

Animal experiments were approved by the Institutional Animal Care and Use Committee of Sun Yat-Sen University Cancer Center, and the animals were cared for in agreement with institutional ethics guidelines. Female BALB/c-nu mice (aged 4 weeks) were purchased from Guangdong Medical Laboratory Animal Center (Guangzhou, China), and injected subcutaneously with either 2×10^6^ HEC-1 cells stably transfected with HK2-shRNA or DLEU2-shRNA into the right flank. Mice were monitored for tumor development and tumor volume was calculated as described previously [2]. To evaluate the anti-tumor effects of TX *in vivo*, HEC-1 cells (2×10^6^) were stably transfected with HK2-shRNA or DLEU2-shRNA and injected subcutaneously into the flanks of nude mice. After the formation of palpable tumors, the mice were further divided into two groups, saline and TX groups. Mice in the TX group received intraperitoneal injection of TX twice a week for 24 days, and tumor volumes were calculated. When mice were sacrificed, the tumor tissues were removed and weighed.

***In vitro* drug studies**

A FAK-specific inhibitor PF-573,228 (PF) was purchased from Santa Cruz Biotechnology. *In vitro* drug studies were performed as previously reported [3, 4, 5]. In brief, Ishikawa cells were treated with 5 µM PF or DMSO for 48 h, and then harvested for western blotting analysis. Confluent monolayers of cells were scratch-wounded using a sterile 200 μl pipette tip, and suspended cells were removed by washing with phosphate-buffered saline twice. The cultures were re-fed with DMEM/F12 media in the presence or absence of PF for 48 h. After 48 h of PF treatment, cells were added to each insert, and allowed to invade for 24 h. For sphere formation assays, PF or DMSO was added into the medium at the beginning of sphere culture.

**Glucose consumption and lactate production assay**

Cells were seeded in culture dishes and the medium was changed after 6 hours. Culture media was collected after incubation for 24 hours. Glucose concentrations in the medium were determined by the Glucose Assay Kit-WST (Dojindo, Kumamoto, Japan). Lactate levels were measured using the Lactate Assay Kit-WST (Dojindo) according to the manufacturer’s instructions. Glucose consumption and lactate production were normalized to cell numbers at the start and end of incubation. Results were presented as fold-change over the respective controls.

**Luciferase reporter assay**

The wild-type (WT) human DLEU2 fragment and the WT *HK2* 3′-untranslated region (3′-UTR) containing the predicted miRNA targeting sites were amplified and cloned into the pGL3-basic vector (Promega, Madison, WI, USA). Mutations (MUT) of the miRNA binding sites in the DLEU2 and *HK2* 3′-UTR were generated using a QuickChange site-directed mutagenesis kit (Stratagene, La Jolla, CA, USA). A luciferase reporter assay was performed as published [1]. In brief, EC cells were seeded in 24-well plates overnight. Then, cells were co-transfected with luciferase plasmids containing DLEU2 (WT or MUT) or *HK2* 3′-UTR (WT or MUT) and miRNA mimic, miRNA inhibitor or the respective control (30 nM) using Lipofectamine 2000 (Invitrogen), together with the Renilla luciferase plasmid pRL-CMV (Promega) used for normalization. After 48 hours, cells were harvested for luciferase detection using the dual-luciferase reporter assay system (Promega). Firefly luciferase activity was normalized against Renilla luciferase activity.

**Subcellular fractionation**

The separation of nuclear and cytoplasmic fractions was performed using the PARIS Kit (Thermo Fisher Scientific, Carlsbad, CA, USA) according to the manufacturer’s instructions. RNA was extracted from both fractions. Then, qRT-PCR analysis was used to evaluate the expression ratios of specific RNA molecules between the cytoplasmic and nuclear fractions, as previously described [6]. U6 served as the nucleus control, and β-actin served as the cytoplasmic control.

**RNA immunoprecipitation (RIP) assay**

RIP experiments were performed using the Magna RIP RNA-Binding Protein Immunoprecipitation Kit (Millipore, Bedford, MA, USA) according to the manufacturer’s instructions. EC cells were collected and lysed using the RIP lysis buffer. 100 μl of cell lysate was incubated with the RIP buffer containing magnetic beads conjugated with antibodies against Argonaute2 (Ago2, #03-110; Millipore), EZH2 (ab3748; Abcam, Cambridge, United Kingdom) or negative control IgG (Millipore). The samples were incubated with Proteinase K (Invitrogen) to digest proteins, and subsequently, immunoprecipitated RNA was isolated. Finally, the purified RNA was subjected to qRT-PCR analysis. *ACTB* mRNA served as the negative control (forward: 5′-CATGTACGTTGCTATCCAGGC-3′; reverse: 5′-CTCCTTAATGTCACGCACGAT-3′).

**Chromatin immunoprecipitation (ChIP) assay**

The ChIP assays were performed using a ChIP kit (#17-371; Millipore) as previously described [7]. Cells were fixed with 1% formaldehyde to crosslink, and nucleoprotein complexes were sheared to 200-500 bases in length with sonication. Then, fifty micrograms of each sonicated chromatin were incubated with 1 µg of antibodies against EZH2 (ab3748; Abcam), H3K27me3 (ab6002; Abcam) or IgG (Millipore) applied as a negative control. The primers used to detect the miR-181a upstream sequence have been previously reported [8].

**Analysis of primary human EC samples and adjacent normal samples**

Fifty primary human EC tissues and the corresponding adjacent normal endometrial tissues were collected from patients, who underwent surgical resection at the Sun Yat-Sen University Cancer Center, China [9]. This study was approved by the Clinical Research Ethics Committee of Sun Yat-Sen University. Informed consent was obtained from all patients. All experiments were performed in accordance with relevant guidelines and regulations. EC and adjacent tissues were snap-frozen in liquid nitrogen immediately after harvesting and stored at -80°C until total RNA was extracted. The qRT-PCR analysis for mRNA and miRNA was performed as described above.

**Statistical analysis**

SPSS 18.0 software (Chicago, IL, USA) was used for statistical analysis. Data are presented as the mean ± standard error of triplicate experiments. Two-tailed Student’s *t*-tests were used to compare the means of two groups, and ANOVA was used to compare the differences among multiple groups. For statistical correlation, Spearman or Pearson correlation coefficient was used when appropriate. Differences with *P*< 0.05 were considered statistically significant.

**References**

1. Konno Y, Dong P, Xiong Y, Suzuki F, Lu J, Cai M, Watari H, Mitamura T, Hosaka M, Hanley SJ, Kudo M, Sakuragi N. MicroRNA-101 targets EZH2, MCL-1 and FOS to suppress proliferation, invasion and stem cell-like phenotype of aggressive endometrial cancer cells. Oncotarget. 2014;5:6049-62.
2. Dong P, Xiong Y, Yue J, Xu D, Ihira K, Konno Y, Kobayashi N, Todo Y, Watari H. Long noncoding RNA NEAT1 drives aggressive endometrial cancer progression via miR-361-regulated networks involving STAT3 and tumor microenvironment-related genes. J Exp Clin Cancer Res. 2019;38(1):295.
3. Juárez-Cruz JC, Zuñiga-Eulogio MD, Olea-Flores M, Castañeda-Saucedo E, Mendoza-Catalán MÁ, Ortuño-Pineda C, Moreno-Godínez ME, Villegas-Comonfort S, Padilla-Benavides T, Navarro-Tito N. Leptin induces cell migration and invasion in a FAK-Src-dependent manner in breast cancer cells. Endocr Connect. 2019;8:1539-1552.
4. Fan H, Guan JL. Compensatory function of Pyk2 protein in the promotion of focal adhesion kinase (FAK)-null mammary cancer stem cell tumorigenicity and metastatic activity. J Biol Chem. 2011;286:18573-82.
5. Waters AM, Stafman LL, Garner EF, Mruthyunjayappa S, Stewart JE, Mroczek-Musulman E, Beierle EA. Targeting Focal Adhesion Kinase Suppresses the Malignant Phenotype in Rhabdomyosarcoma Cells. Transl Oncol. 2016;9:263-73.
6. Shan Y, Ma J, Pan Y, Hu J, Liu B, Jia L. LncRNA SNHG7 sponges miR-216b to promote proliferation and liver metastasis of colorectal cancer through upregulating GALNT1. Cell Death Dis. 2018;9:722.
7. Chen Q, Cai J, Wang Q, Wang Y, Liu M, Yang J, Zhou J, Kang C, Li M, Jiang C. Long Noncoding RNA NEAT1, Regulated by the EGFR Pathway, Contributes to Glioblastoma Progression Through the WNT/β-Catenin Pathway by Scaffolding EZH2. Clin Cancer Res. 2018;24:684-695.
8. Cao Q, Mani RS, Ateeq B, Dhanasekaran SM, Asangani IA, Prensner JR, Kim JH, Brenner JC, Jing X, Cao X, Wang R, Li Y, Dahiya A, Wang L, Pandhi M, Lonigro RJ, Wu YM, Tomlins SA, Palanisamy N, Qin Z, Yu J, Maher CA, Varambally S, Chinnaiyan AM. Coordinated regulation of polycomb group complexes through microRNAs in cancer. Cancer Cell. 2011;20:187-99.
9. Dong P, Xiong Y, Yue J, Hanley SJB, Watari H. miR-34a, miR-424 and miR-513 inhibit MMSET expression to repress endometrial cancer cell invasion and sphere formation. Oncotarget. 2018;9:23253-23263.

**Supplemental Figure 1-12**

**
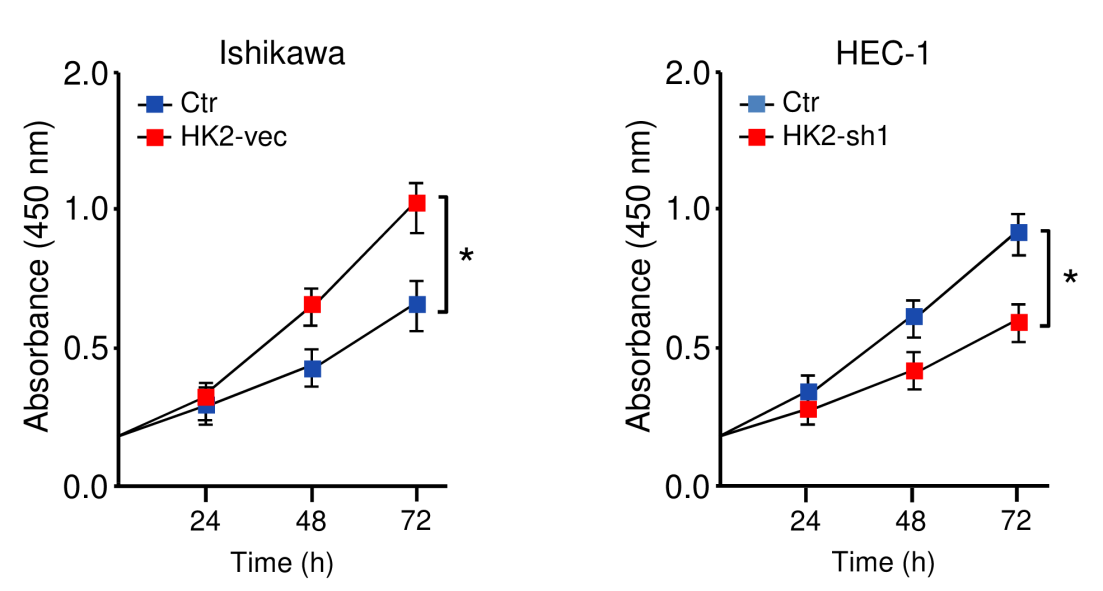
**

**Supplemental Figure 1**: HK2 promotes the proliferation of EC cells.

Growth curves of EC cells transfected as indicated were determined using Cell Counting Kit-8 assays. Vec: vector; sh1: shRNA-1. **P*<0.05.

**
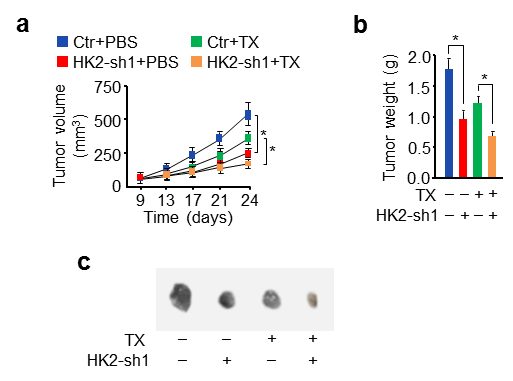
**

**Supplemental Figure 2**: HK2 facilitates *in vivo* cell proliferation and chemoresistance of EC.

(**a, b**) Growth curves (**a**) and quantification of the weight (**b**) of subcutaneous control or HK2-silenced HEC-1 xenografts treated with vehicle or TX are shown. (**c**) Representative images of tumor xenografts derived from nude mice. **P*<0.05.


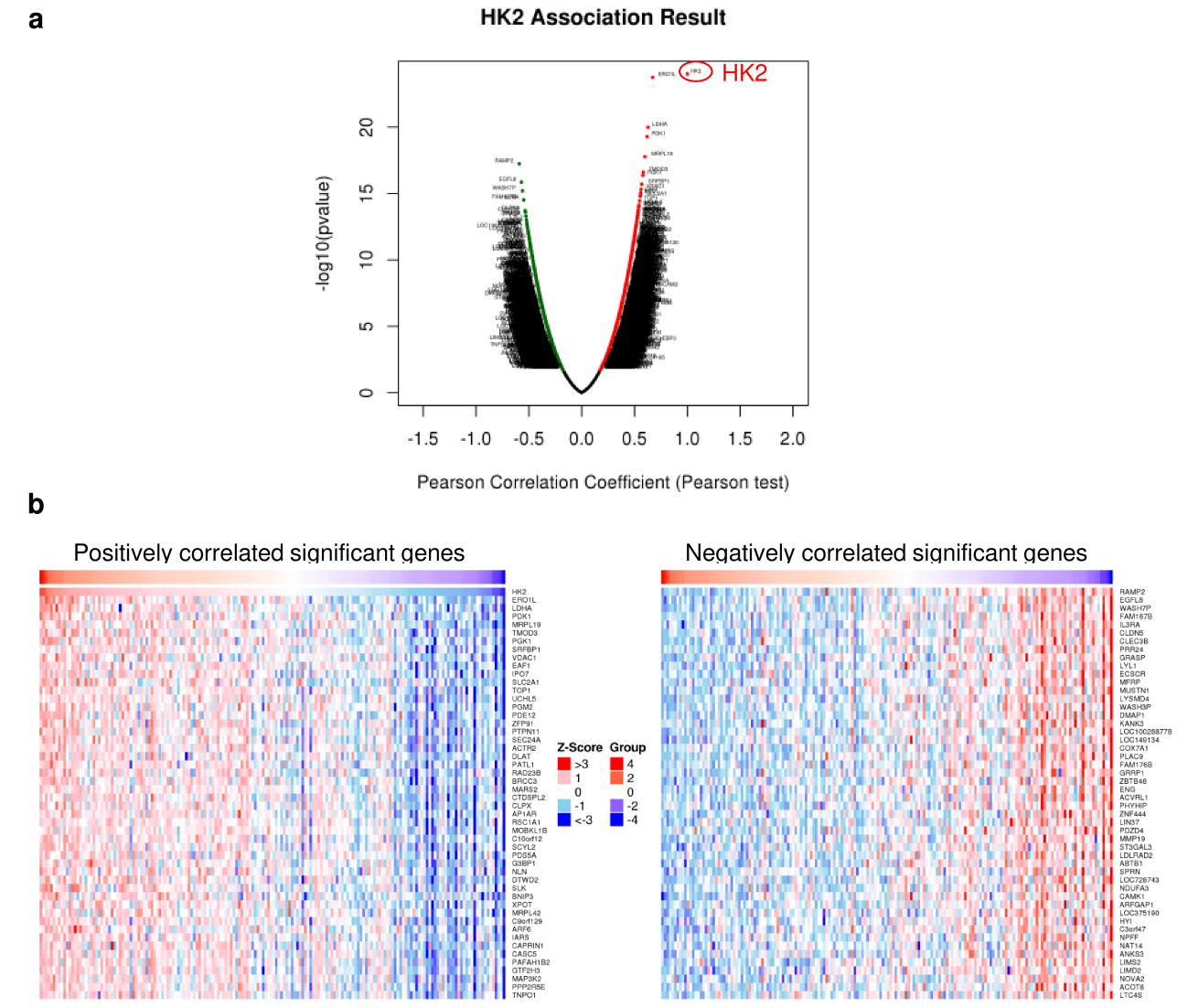


**Supplemental Figure 3**: Genes highly co-expressed with HK2 in EC tissues.

(**a**) Genes highly co-expressed with HK2 in TCGA EC dataset from the LinkedOmics were selected. (**b**) Heat map of positively and negatively correlated genes with HK2 in EC tissues.


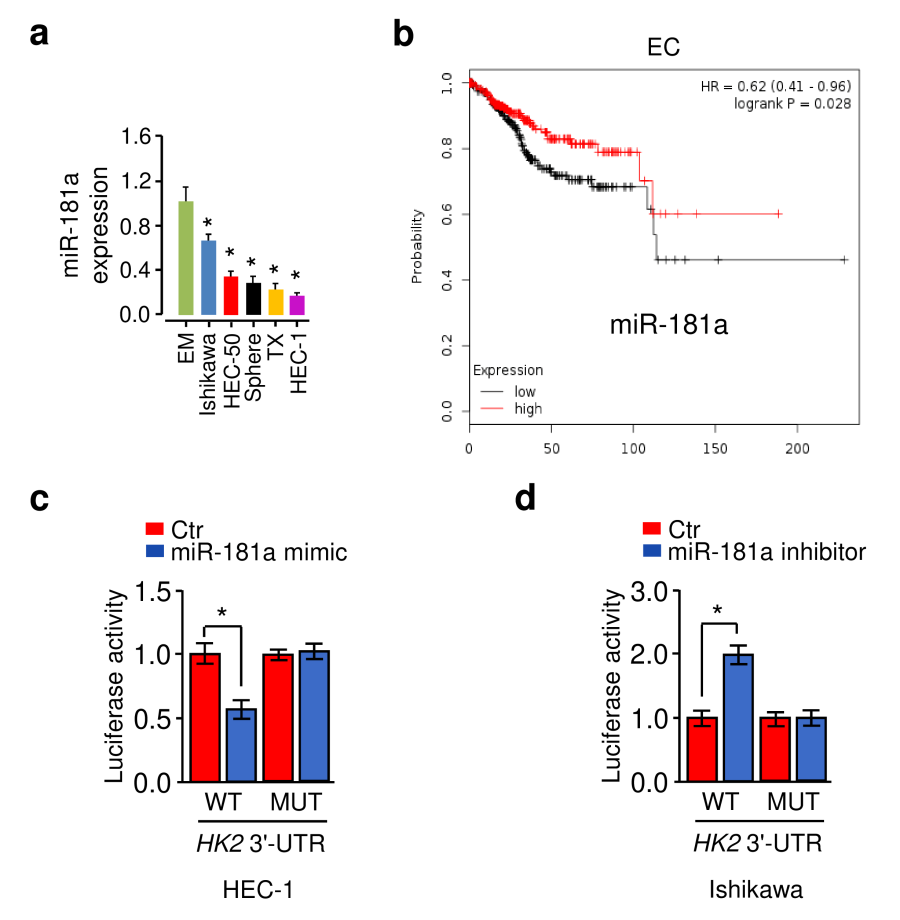


**Supplemental Figure 4**: MiR-181a directly targets HK2 in EC cells.

(**a**) qRT-PCR analysis of miR-181a expression in normal EM and EC cells. (**b**) Kaplan-Meier overall survival analysis was used to assess EC patients with high or low miR-181a expression based on the TCGA data with KM Plotter. (**c**, **d**) Luciferase reporter assay with HEC-1 cells co-transfected with a luciferase reporter plasmid containing WT or mutant (MUT) *HK2* 3′-UTR, along with miR-181a mimic or control mimic (**c**), and with Ishikawa cells co-transfected with a luciferase reporter plasmid containing WT or mutant *HK2* 3′-UTR, along with miR-181a inhibitor or control inhibitor (**d**). **P*<0.05.

**
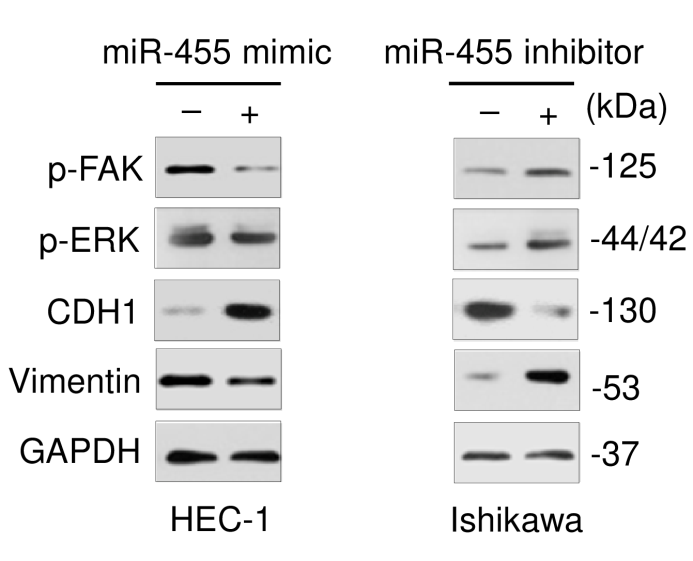
**

**Supplemental Figure 5**: Effects of miR-455 on the expression of HK2 downstream effectors in EC cells.

Western blotting analysis of the indicated proteins in EC cells following overexpression or knockdown of miR-455.


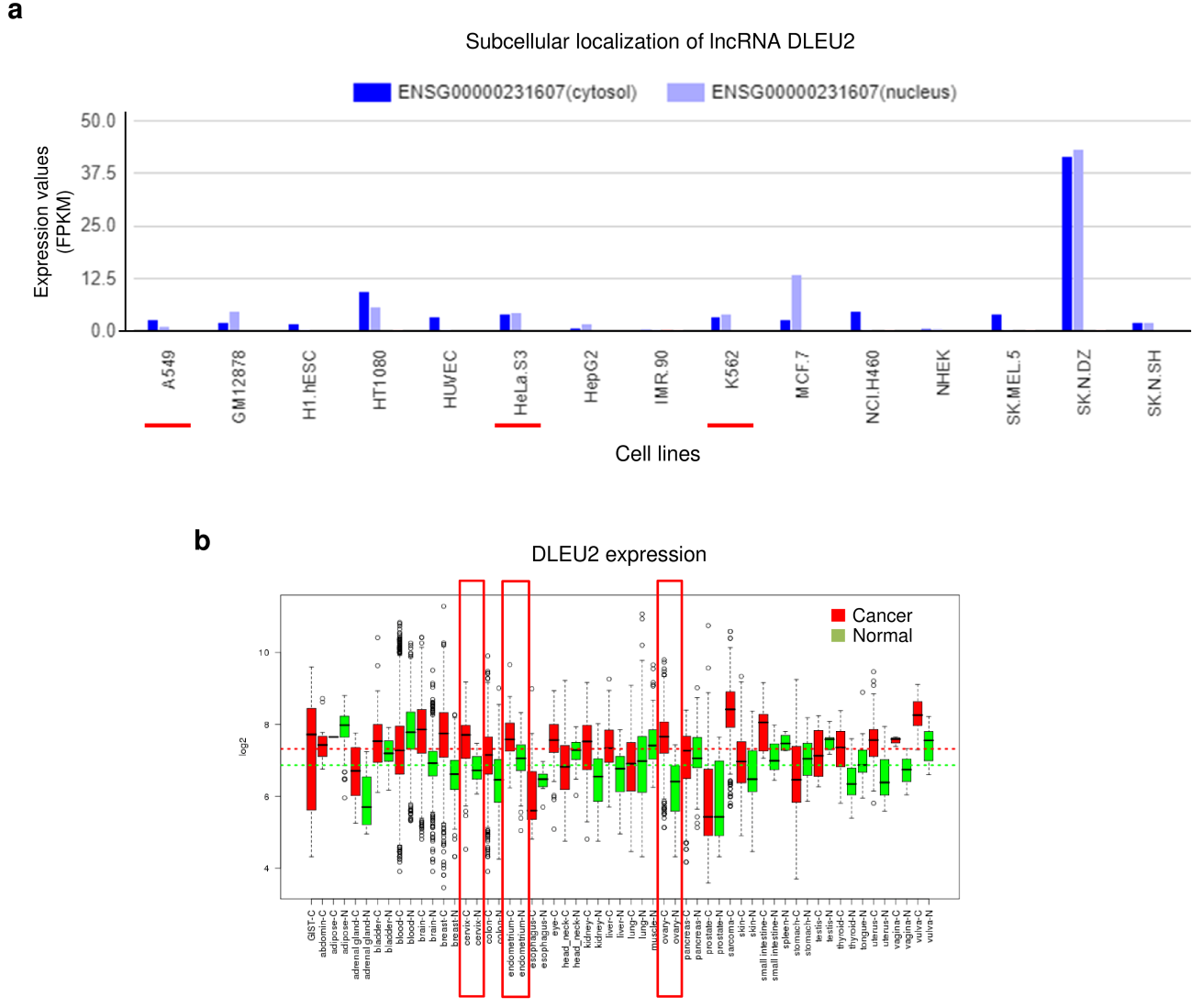


**Supplemental Figure 6**: Subcellular localization and expression pattern of lncRNA DLEU2 in human tumors.

(**a**) Subcellular localization of lncRNA DLEU2 in human tumor cells (lncATLAS database). (**b**) The expression pattern of DLEU2 in normal and tumor tissues was searched using the GENT database. Red boxes indicate a significant increase in DLEU2 expression in endometrial, cervical, and ovarian cancer tissues compared with the respective normal tissues.


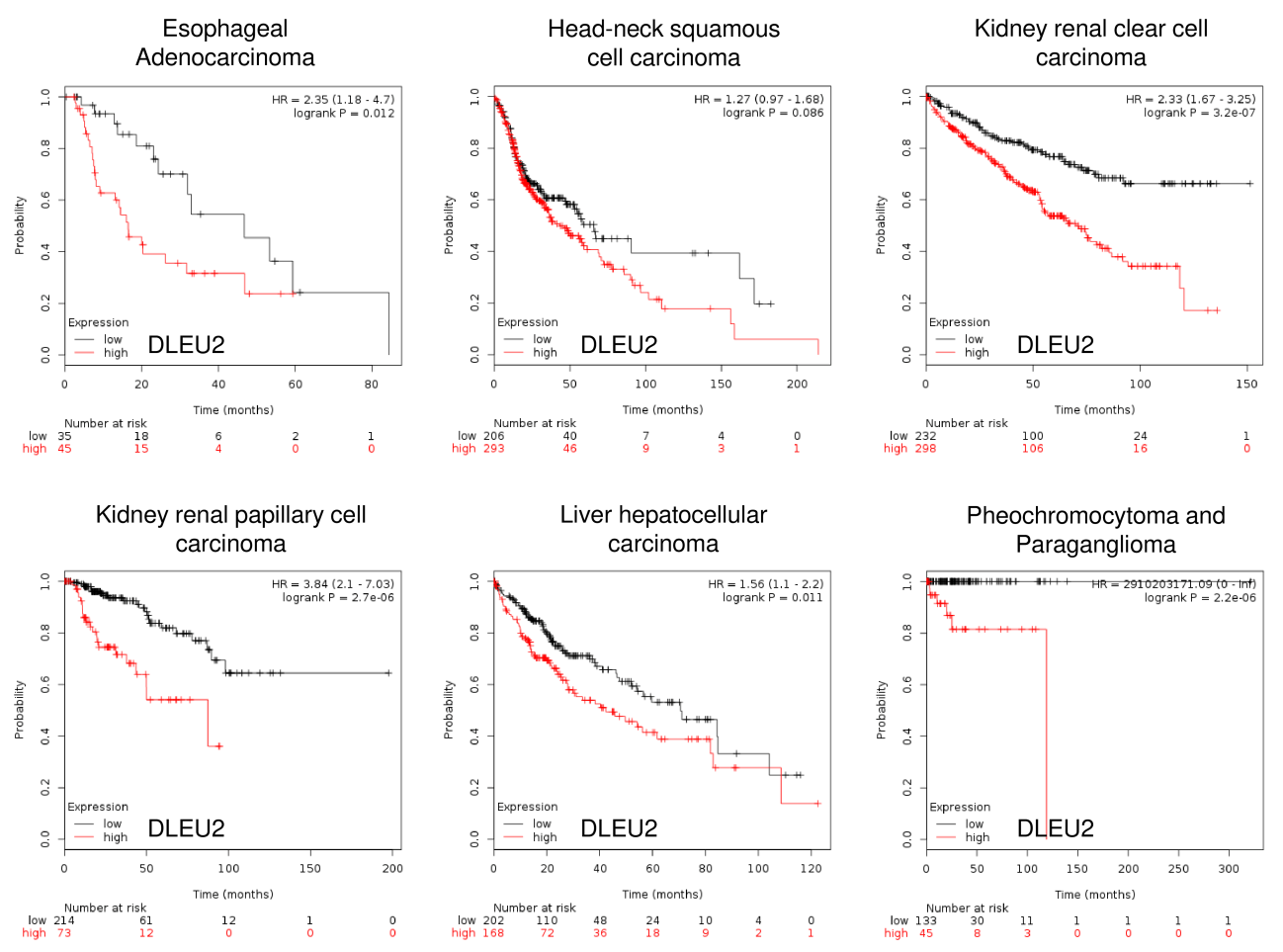


**Supplemental Figure 7**: The prognostic value of DLEU2 in human tumors.

The survival curves comparing patients with high (red) and low (black) DLEU2 expression in esophageal, head-neck, kidney, liver cancers, and pheochromocytoma/paraganglioma were plotted from the KM Plotter database.


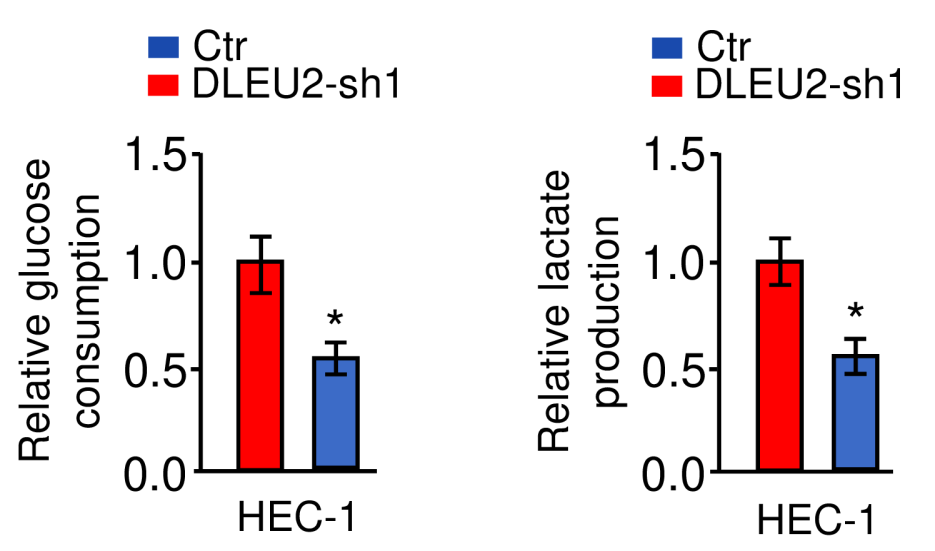


**Supplemental Figure 8**: DLEU2 drives glycolysis in EC.

Cell glucose consumption and lactate production of HEC-1 cells following knockdown of DLEU2. Sh1: shRNA-1. **P*<0.05.


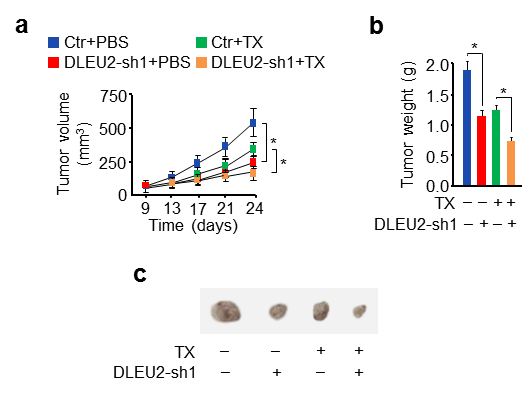


**Supplemental Figure 9**: DLEU2 enhances *in vivo* cell proliferation and chemoresistance of EC.

(**a**, **b**) Growth curves (**a**) and quantification of the weight (**b**) of subcutaneous control or DLEU2-silenced HEC-1 xenografts treated with vehicle or TX are shown. (**c**) Representative images of tumor xenografts derived from nude mice **P*<0.05.


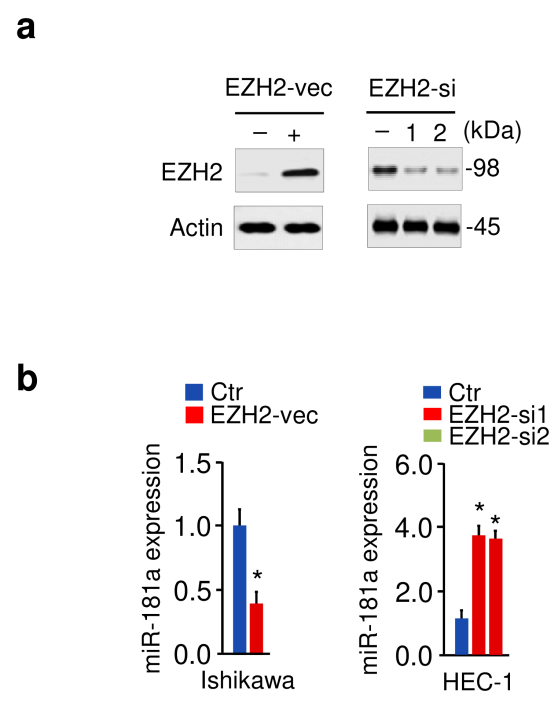


**Supplemental Figure 10**: EZH2 inhibits miR-181a expression in EC cells.

(**a**) Western blotting analysis of the indicated proteins in EC cells following overexpression or knockdown of EZH2. (**b**) qRT-PCR analysis of miR-181a expression in EC cells following overexpression or knockdown of EZH2. Vec: vector; si: siRNA. **P*<0.05.


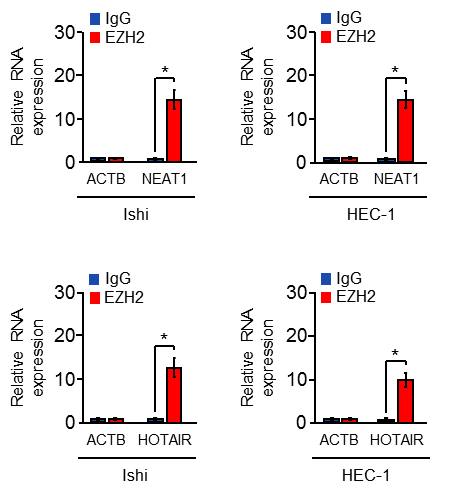


**Supplemental Figure 11:** RIP-qPCR validated the interaction of EZH2 with two known EZH2-interactor lncRNAs (NEAT1 and HOTAIR) in Ishikawa and HEC-1 cells. *ACTB* was used as the negative control. **P*<0.05.


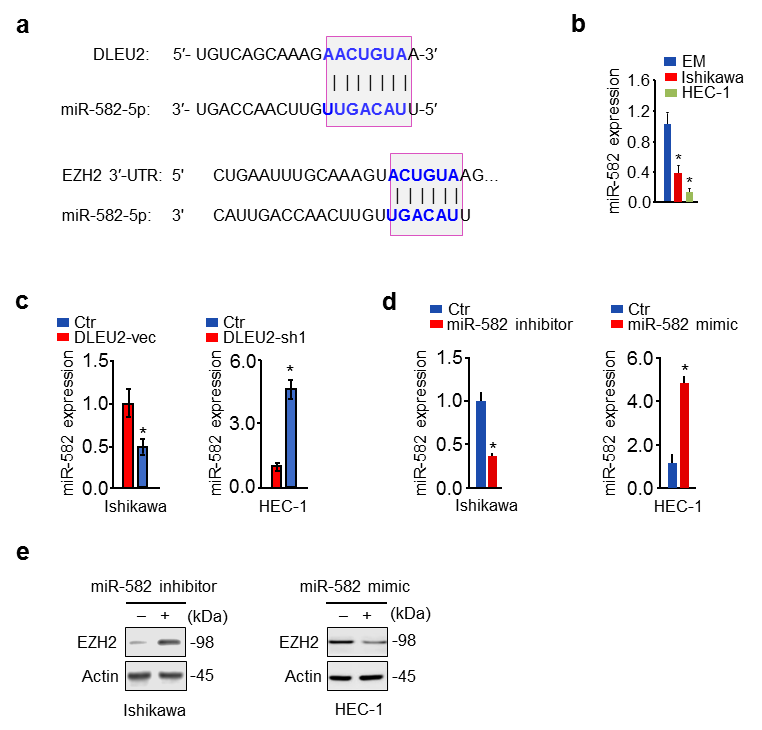


**Supplemental Figure 12**: DLEU2-mediated miR-582 repression induces EZH2 expression in EC cells.

(**a**) The putative binding sites for miR-582 in the DLEU2 and *HK2* 3′-UTR sequence. (**b**) qRT-PCR analysis of miR-582 levels in EM and EC cells. (**c**) The expression of miR-582 in EC cells following overexpression or knockdown of DLEU2. (**d**) The levels of miR-582 in EC cells upon overexpression or knockdown of miR-582. (**e**) Western blotting analysis of the indicated proteins in EC cells following overexpression or knockdown of miR-582. Vec: vector; sh1: shRNA-1. **P*<0.05.
